# Fluorogenic PNA probes

**DOI:** 10.3762/bjoc.14.17

**Published:** 2018-01-29

**Authors:** Tirayut Vilaivan

**Affiliations:** 1 Organic Synthesis Research Unit, Department of Chemistry, Faculty of Science, Chulalongkorn University, Phayathai Road, Patumwan, Bangkok 10330, Thailand

**Keywords:** DNA, fluorescence, molecular beacons, molecular probes, oligonucelotides, RNA

## Abstract

Fluorogenic oligonucleotide probes that can produce a change in fluorescence signal upon binding to specific biomolecular targets, including nucleic acids as well as non-nucleic acid targets, such as proteins and small molecules, have applications in various important areas. These include diagnostics, drug development and as tools for studying biomolecular interactions in situ and in real time. The probes usually consist of a labeled oligonucleotide strand as a recognition element together with a mechanism for signal transduction that can translate the binding event into a measurable signal. While a number of strategies have been developed for the signal transduction, relatively little attention has been paid to the recognition element. Peptide nucleic acids (PNA) are DNA mimics with several favorable properties making them a potential alternative to natural nucleic acids for the development of fluorogenic probes, including their very strong and specific recognition and excellent chemical and biological stabilities in addition to their ability to bind to structured nucleic acid targets. In addition, the uncharged backbone of PNA allows for other unique designs that cannot be performed with oligonucleotides or analogues with negatively-charged backbones. This review aims to introduce the principle, showcase state-of-the-art technologies and update recent developments in the areas of fluorogenic PNA probes during the past 20 years.

## Review

### Introduction

The development of molecular probes that can detect and quantify specific biological molecules with a high degree of sensitivity and accuracy, preferably in situ and in real-time, is an important research area since it contributes to the advancement of understanding of complex biomolecular systems and has practical applications in diverse areas, including clinical diagnostics and drug discovery amongst others. Molecular probes generally consist of a recognition element that can bind to the specific target, and a reporter group that, in combination with an appropriate signal transduction mechanism, translates the molecular interaction into a measurable signal. Among several available modes of detection, fluorescence detection offers a number of distinct advantages, but mainly that it is relatively simple, selective, highly sensitive and can be used for real-time monitoring of the biological targets and even their interactions in vivo. For nucleic acids, the latter valuable information cannot be obtained by sequencing despite the tremendous advances in the field in recent years [1].

**Figure 1:**
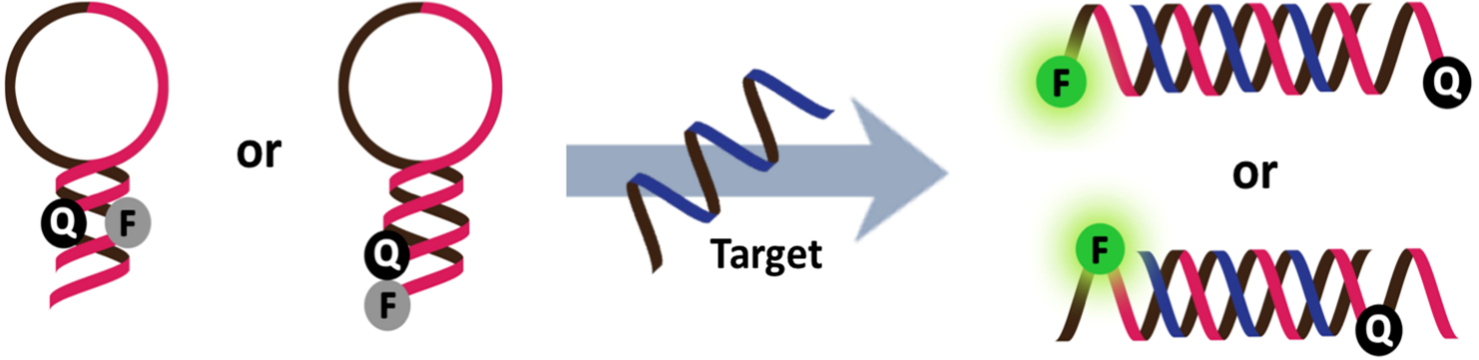
The design of classical DNA molecular beacons.

Accordingly, fluorescent oligonucleotide probes are still one of the most important tools not only for the detection of nucleic acids, but also proteins and other non-nucleic acid targets by employing aptamer technology [2]. However, ordinary fluorescent oligonucleotide probes generally show indistinguishable signals between the free and target-bound states. This means that additional treatments are required in order to separate the bound and unbound probes, which is most commonly achieved by solid phase hybridization followed by washing to eliminate the unbound probes before performing the fluorescence readout. These assays require multiple steps, and so are time-consuming making them unsuitable for real-time monitoring, such as nucleic acid amplification, monitoring of enzyme activities and localization and quantitation of nucleic acids in living cells. Therefore, it is highly desirable to develop a “smart” fluorogenic oligonucleotide probe that can directly report the presence of the target by a simple fluorescence readout in homogeneous and wash-free format. One of the landmark developments in this area is the DNA molecular beacon – a double-labeled hairpin DNA that can change its fluorescence in response to the conformational change induced by binding with the complementary nucleic acid target (Figure 1) [3–4].

While a number of signal transduction and readout strategies have been developed, even for fluorescence-based detection alone, the recognition element of the probe is almost always an oligodeoxynucleotide. During the past few decades, there have been continuous developments of unnatural oligonucleotides with superior properties that make them worthwhile alternatives to the traditionally used oligodeoxynucleotide probes. In this respect, peptide nucleic acids (PNA) [5] have been one of the most widely used alternative oligonucleotide probes in addition to locked and morpholino nucleic acids [6–7]. Although many review articles on fluorogenic oligonucleotide probes exist [8–17], very few have specifically dealt with fluorogenic unnatural oligonucleotide probes [18–19]. This review aims to fill this gap by introducing the principle, showcasing state-of-the-art technologies and updating recent developments in the areas of fluorogenic PNA probes. Some examples of PNA probes for nucleic acid hybridization and detection that may not be strictly fluorogenic by definition have been included where deemed suitable.

### Overview of PNA and fluorescent PNA probes

Peptide nucleic acids (PNA) are a unique class of oligonucleotide mimics that consist of a peptide-like backbone. Although the idea of replacing the whole sugar-phosphate backbone of DNA with a completely unrelated scaffold such as peptide had been around since the 1970s [20], it was not until the 1990s that the first PNA system with an *N*-2-aminoethylglycine (aeg) backbone that can recognize its target DNA and RNA was reported [21]. Considering the enormous difference between the two backbones, it is quite surprising that PNA can still retain the ability to recognize natural oligonucleotides having a complementary sequence with high affinity and specificity according to the Watson–Crick base pairing rules [22]. In fact, PNA exhibits an even higher affinity and better discrimination between complementary and mismatched nucleic acid targets than natural oligonucleotides. In addition, the uncharged peptide-like backbone of PNA contributes to several unique properties not observed in other classes of oligonucleotide analogues with negatively charged phosphate groups. These include the relative insensitivity of the PNA–DNA or PNA–RNA hybrids to the ionic strength of the solvent [23], and the complete stability towards nucleases as well as proteases [24].

**Figure 2:**
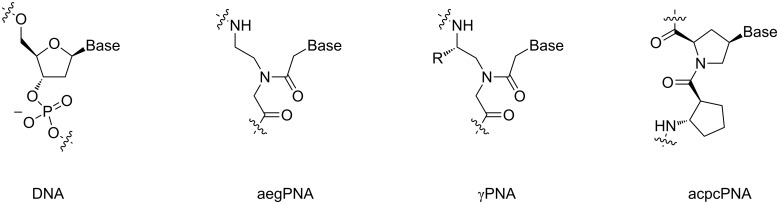
Structures of DNA and selected PNA systems.

Overall, these properties enable the use of PNA for several applications, including therapeutics and diagnostics [25], and as a unique tool for DNA manipulation, such as PCR clamping [26] and PNA openers [27]. Ironically, the very same uncharged nature of PNA that is the basis of several of the aforementioned advantages poses new challenges, including a poor aqueous solubility and the tendency to bind non-specifically to hydrophobic surfaces, including other PNA molecules to form aggregates. These can be largely overcome by conjugation of the PNA to charged or hydrophilic groups or by backbone modification [28]. The simplicity of the PNA structure offers unlimited possibilities to design new PNA systems with improved solubilities and other properties and to incorporate new functions into the PNA molecule [29]. Some notable performance improvements of the original PNA system based on the aeg backbone have been realized by constraining the conformational flexibility by incorporating a suitable substituent at a suitable position (such as in γPNA) [30], or by incorporating cyclic structures into the PNA backbones (such as in acpcPNA, Figure 2) [31–32].

**Figure 3:**
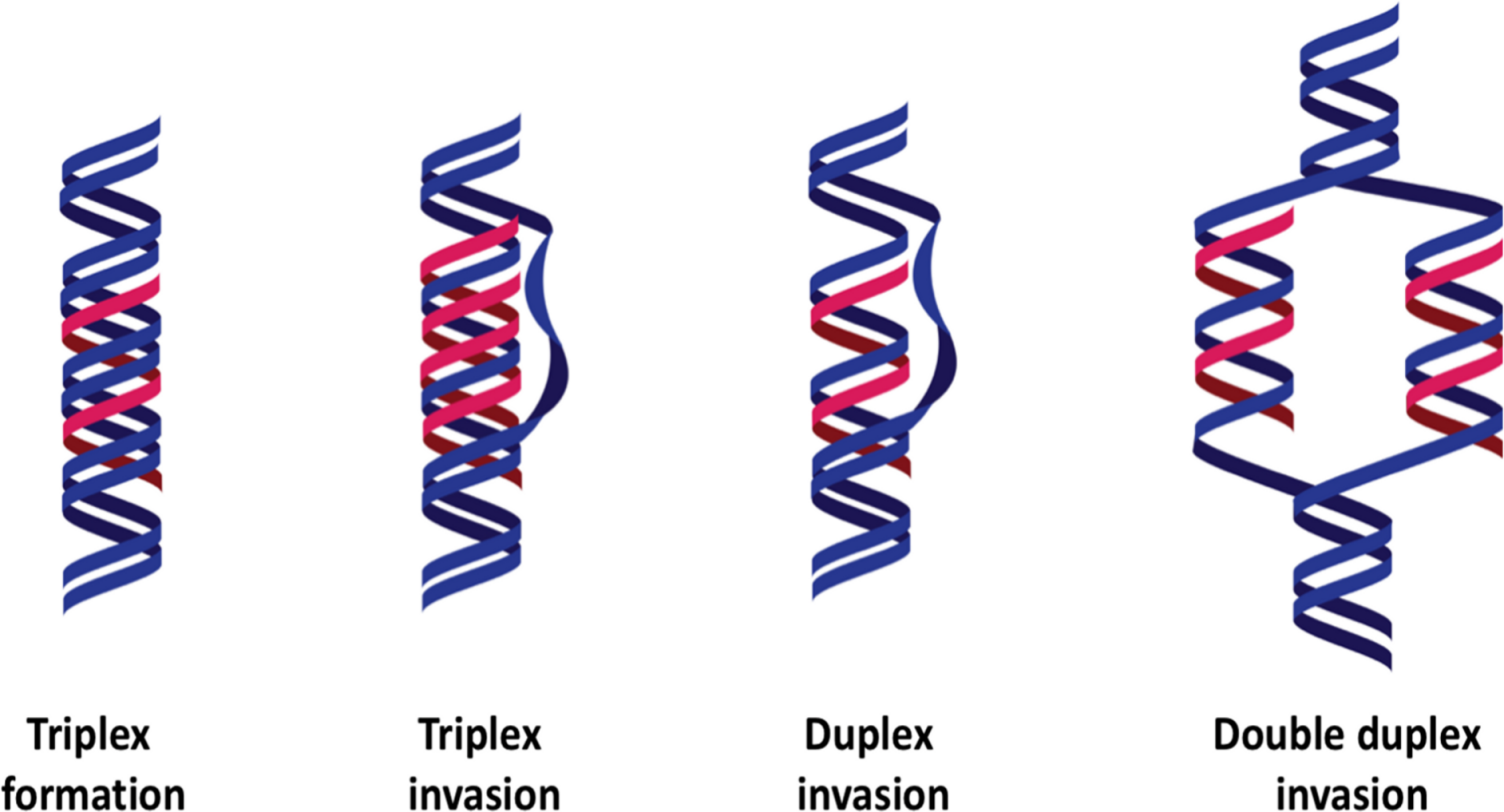
Various binding modes of PNA to double stranded DNA including triplex formation, triplex invasion, duplex invasion and double duplex invasion.

The high binding affinity and excellent specificity of PNA towards their respective nucleic acid targets immediately suggested their potential use as diagnostic probes. In addition, a broad range of salt tolerance and stability against most enzymes are added benefits of PNA probes. Moreover, the ability of PNA to bind to double stranded (ds)DNA (Figure 3) [33], dsRNA [34], and other unusual structures, such as G-quadruplexes [35], makes PNA an ideal tool for targeting structured nucleic acid targets. Simple fluorescent-labeled PNA probes have found extensive applications in nucleic acid detection and quantitation. Examples of such assays that have successfully employed PNA probes include array hybridization [36–37] and staining of intracellular nucleic acids by fluorescence in situ hybridization (FISH) [38–39]. For the same reasons explained above in the case of oligonucleotide probes, it is more desirable to develop a fluorogenic PNA probe that can change its fluorescence signal in response to hybridization to its specific target. The most obvious strategy would be to employ the same principle as DNA beacons, whereby two interacting dyes are placed on a hairpin-forming PNA probe that can switch to an open conformation upon target hybridization [3–4].

It has been reported that short, linear PNA beacons can perform surprisingly well when compared to linear DNA beacons [40]. This could be attributed to both the ability of PNA to form a compact structure in aqueous media, thereby forcing the two dyes in close contact, and the excellent mismatch discrimination ability of PNA. Several other strategies originally developed for fluorogenic oligonucleotide probes have also been successfully applied to PNA probes. These include binary probes and nucleic acid-templated reactions, strand displacement probes and the combination of ordinary PNA probes with nanomaterials as an external quencher. In addition, the uncharged backbone of PNA offers other unique designs, including the combination of PNA probes with cationic conjugated polymers that simultaneously act as a light harvesting antenna and fluorescent resonance energy transfer (FRET) partner, and the combination of short linear PNA probes with environment-sensitive dyes, such as in light-up and forced intercalation (FIT) PNA probes.

#### PNA probes carrying two or more interacting dyes in the same strand

**Figure 4:**
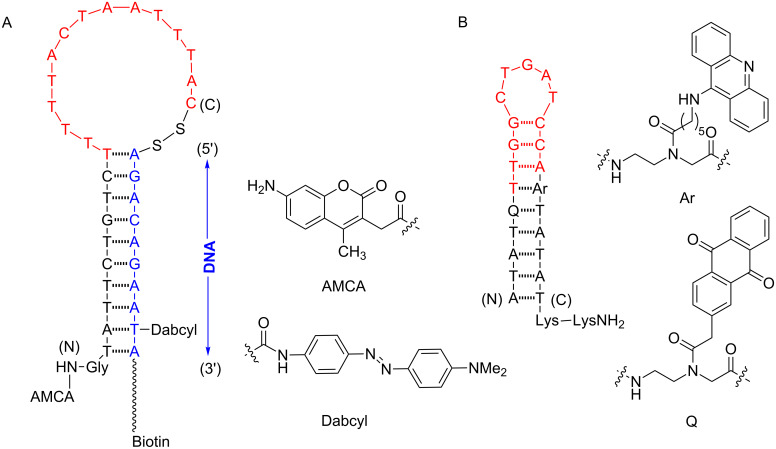
The design and working principle of the PNA beacons according to (A) Ortiz et al. [41] and (B) Armitage et al. [42]. The DNA binding domains are shown in red.

The very first PNA probe of this type were independently reported in 1998 by Ortiz et al. [41] and Armitage et al. [42]. These first-generation PNA beacons employed a similar design principle to that of classical DNA beacons, having a hairpin structure carrying a fluorophore and a quencher attached to the stem part. In the normal state, the adopted hairpin structure forced the fluorescence dye and quencher to be in close proximity, resulting in quenching of the dye. Binding to the complementary DNA target opened the hairpin structure and restored the fluorescence. In Armitage’s design [42], the entire beacon was purely PNA, while in the Ortiz design [41], PNA was employed as the DNA binding domain and the stem part consisted of a chimeric PNA–DNA hybrid linked together by a disulfide bond (Figure 4). The chimeric beacon was immobilized to a microtiter plate via a biotin tag and was employed for the detection of PCR amplicons of rDNA from *Entamoeba histolytica* [41]. The use of PNA allowed direct detection of double stranded (ds)DNA targets after simple denaturation due to the ability of PNA to bind more strongly to DNA and so effectively compete with the DNA re-association.

Another research group from Japan also reported a similar stem-loop non-chimeric PNA beacon carrying two labels (TAMRA/Dabcyl) at both ends of the PNA molecule and demonstrated that the fluorescence changed in response to the presence of the DNA target [43]. The high stability of PNA–PNA self-hybrids allows the design of a beacon with a relatively short stem (four bases). In this particular case, an interesting pH dependency of the performance of the PNA beacon was observed, but no explanation was offered.

**Figure 5:**
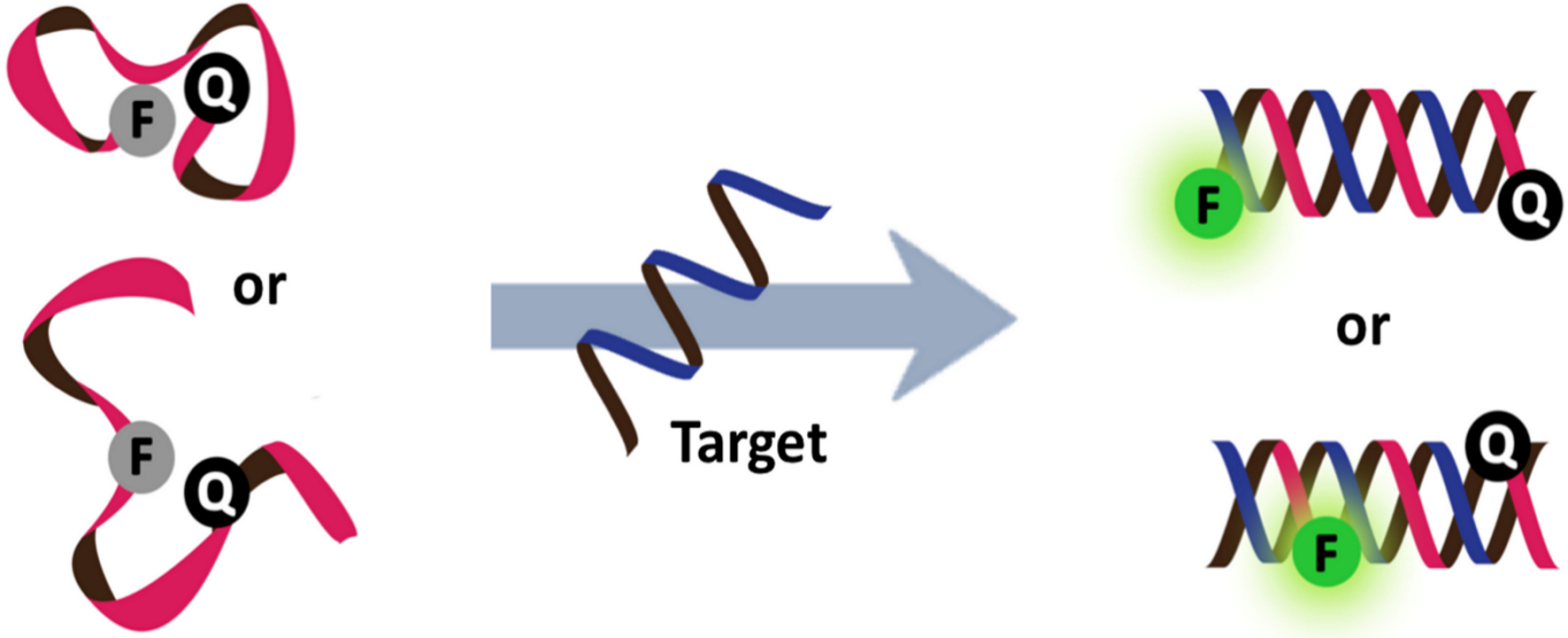
The design of "stemless" PNA beacons.

**Figure 6:**
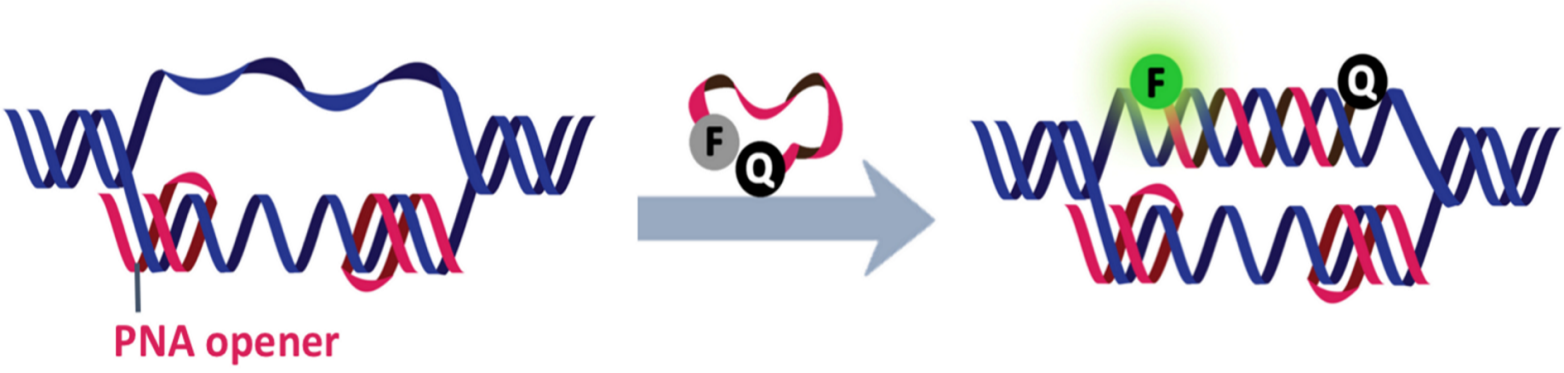
The applications of PNA openers to facilitate the binding of PNA beacons to double stranded DNA [40,47].

A new generation of PNA beacons was simultaneously reported by Seitz [44–45] and a group at Boston Probes [40,46]. According to this design, a short linear PNA strand was double-labeled at both ends with a quencher and a fluorophore (Figure 5). Seitz proposed that the stem-loop structure was not required because the hydrophobic backbone of PNA tends to adopt a compact structure to minimize hydrophobic surfaces, allowing the fluorophore and quencher labeled at opposite ends of the PNA molecule to make a close contact. However, based on their detailed comparative studies between stemless PNA and DNA beacons, the Boston Probes group argued that the quenching was more likely due to hydrophobic and electrostatic interactions between the fluorophore and the quencher. Nevertheless, different fluorophore-quencher pairs were used in the two cases, and so no single explanation may be applicable to all circumstances. What is clear is that the stemless, unstructured linear PNA beacons showed an impressive performance in DNA detection in terms of an excellent specificity, very fast hybridization kinetics and high signal-to-background ratio regardless of the salt concentration. Moreover, it can be applied for the direct detection of dsDNA targets under non-denaturing conditions either directly for short dsDNA sequences [47] or with the assistance of a pair of PNA openers for longer dsDNA targets (Figure 6) [40].

**Figure 7:**
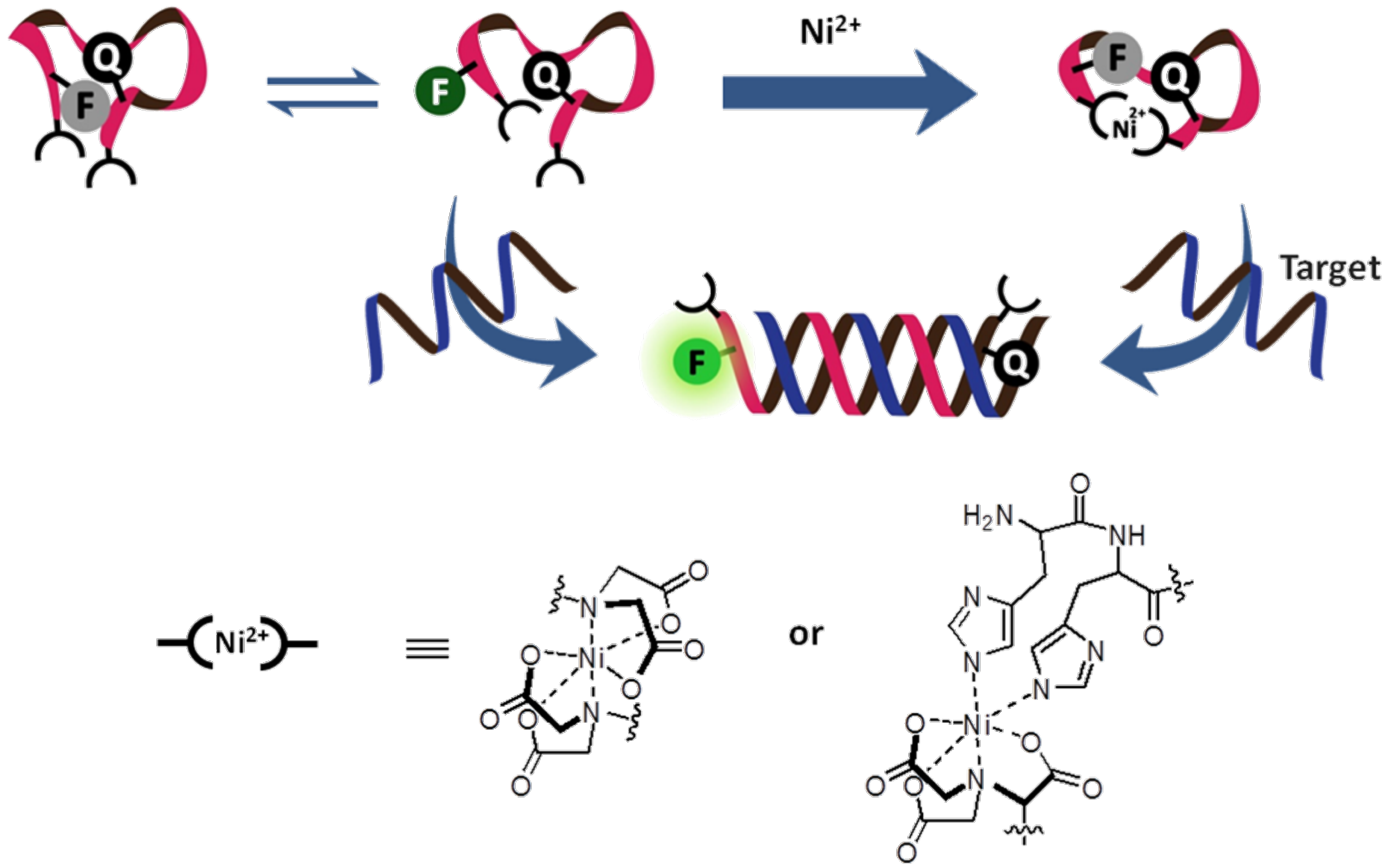
The working principle of snap-to-it probes that employed metal chelation to bring the dyes in close contact and to improve the mismatch discrimination by increasing the thermodynamic barrier of the probe–target binding [48].

To ensure a close contact between the two labels, amino acids with opposite charges (typically glutamic acid and lysine) are usually placed in the vicinity of the labels. This was the practice originally used by the Boston Probe design and has been followed since by many others [40]. In another strategy, the PNA may be terminally functionalized with chelating ligands, such as iminodiacetic acid, iminotriacetic acid or terminal dihistidine, to form the so-called Snap-to-it probes (Figure 7) [48]. The addition of divalent metal ions, such as Ni^2+^, results in an intramolecular chelation that contributes to the reduction in the background fluorescence of the single stranded (ss) probe by forcing the labels in close contact. In addition, the chelation also introduces an additional thermodynamic barrier into the binding of the DNA target to the unstructured PNA beacons that is akin to the difference observed between linear and hairpin DNA beacons [49]. These result in a significant (up to 40-fold) improvement in the signal-to-background ratio and target binding specificity [48].

A different strategy to eliminate the background signal problem from free PNA beacons is to use ion exchange HPLC with fluorescence detection which can separate the signals from the free and hybridized PNA probes into different channels. In this HPLC-based assay, the selectivity for single mismatch detection was greatly improved compared to the homogeneous assay using the same probe and targets, which is probably due to the denaturing effects of the chromatographic conditions employed [50–51].

**Figure 8:**
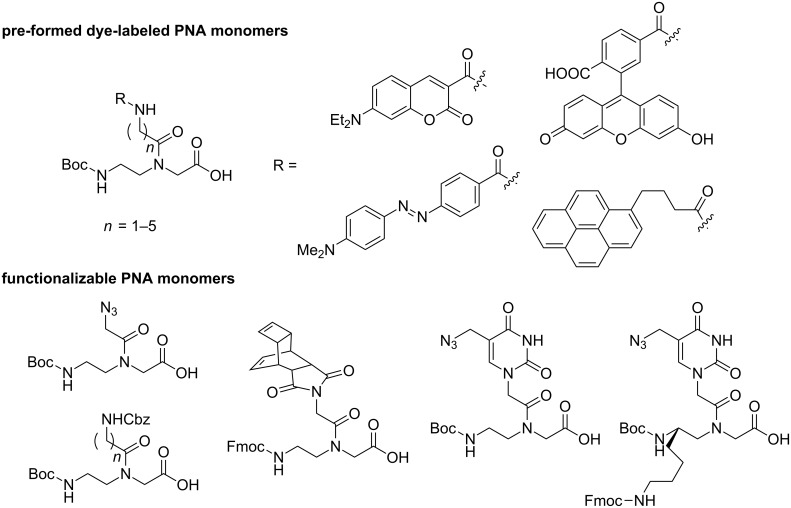
Examples of pre-formed dye-labeled PNA monomers and functionalizable PNA monomers.

Most dual-labeled PNA beacons discussed so far carry a fluorophore and a quencher attached at opposite ends of the PNA strand via amide bonds. The advantage of this strategy is that the standard as-synthesized PNA can be used directly without having to modify the synthetic protocols, but it is difficult to vary the positions of the label attachment. The use of pre-formed dye labeled PNA monomers [42,52–53] or post-synthetically functionalizable PNA monomers [54–59] (Figure 8) permits facile incorporation of the labels anywhere in the molecular beacon, which may allow better control of the interactions between the two dyes and is, therefore, an important step towards a further improvement in the performance of the PNA probes.

**Figure 9:**
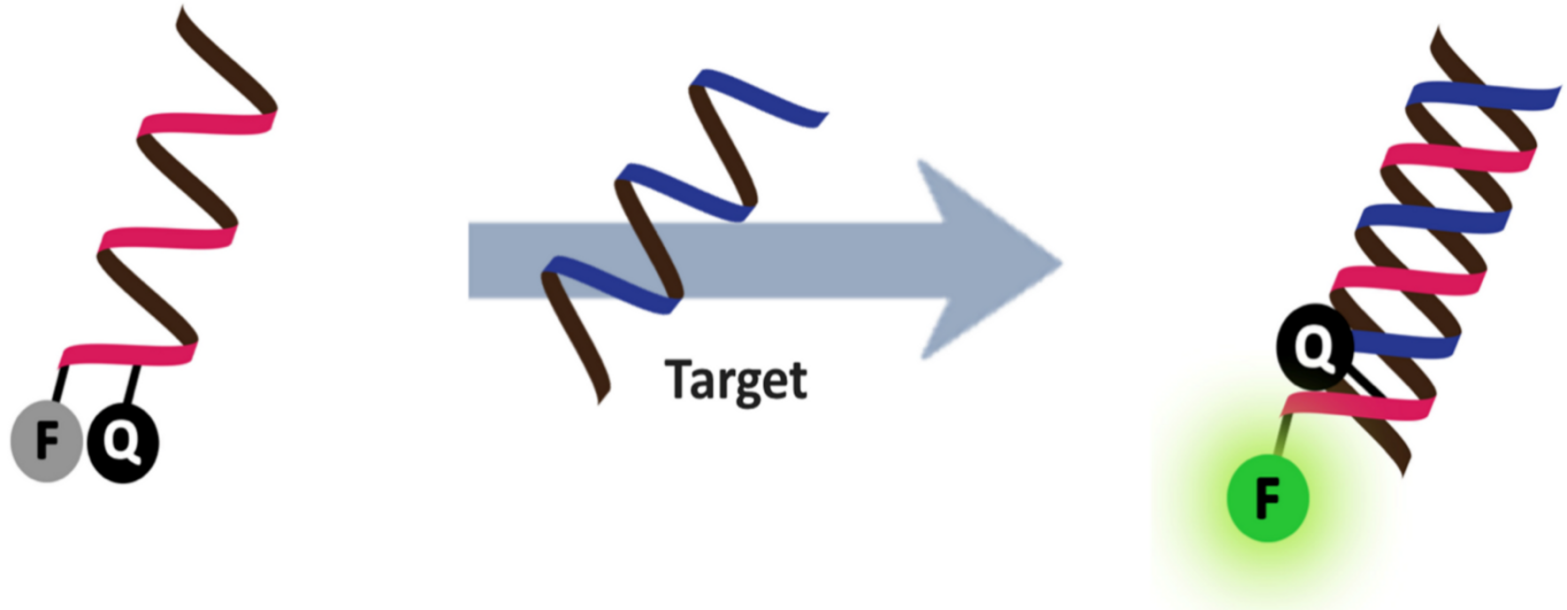
Dual-labeled PNA beacons with end-stacking or intercalating quencher.

In an alternative design, the fluorophore and either the quencher or another fluorophore may be placed in close proximity in the probe molecule, thereby maximizing the interactions between the two dyes and leading to a more effective quenching. Duplex formation will alter the interaction if one of the dyes can intercalate into the duplex or form a more stable end-stacking complex with the terminal base pair of the duplex (Figure 9). Several examples of DNA probes with this design are known [60–64]. This concept has been applied in the design of a double-end-labeled conformationally constrained acpcPNA probe for DNA sequence detection that showed a low background and good response with DNA, as well as offering an excellent specificity [65].

In addition to fluorophore-quencher interactions, FRET [66] and pyrene monomer–excimer switching [67–68] have been employed as alternative mechanisms for inducing fluorescence changes in the DNA probes. The FRET and monomer–excimer switching approaches have some advantages over fluorescence quenching because of the large Stokes shifts and the ability to measure the signals at two different wavelengths, thereby providing a means for self-referencing. Furthermore, unlike the fluorophore-quencher beacons, the FRET and monomer–excimer switching beacons are also fluorescent in the unbound state, and therefore it is possible to monitor the success of cellular delivery. In the case of pyrene monomer–excimer switching, the long fluorescence lifetime of the pyrene excimers allows facile elimination of background signals from autofluorescence by time-resolved fluorescence measurements. A few monomer–excimer switching PNA probes have been reported. Two or more pyrene labels may be conveniently placed anywhere in the PNA molecule by attaching them to a C5-functionalized thymine via amide or click chemistries, and applications for the detection of DNA by duplex [54] or triplex [69] formation have been demonstrated. Alternatively, the pyrene label can be placed onto the PNA backbone as a base replacement (in aegPNA) [55] or as a tethered label through a flexible linker (in acpcPNA) [70].

In most cases, excimer emission is predominant in the single stranded probe, and hybridization with the DNA target resulted in an increased monomer emission with a simultaneous decrease in the excimer emission. A switching ratio of >30 upon hybridization with the correct DNA target was obtained with double-pyrene-labeled acpcPNA probes [70]. Interestingly, switching in the opposite direction, when the excimer signal is enhanced upon hybrid formation, was observed in triplex forming double-pyrene-labeled aegPNA probes [69]. For FRET-based PNA beacons, these have so far been investigated in the context of FIT PNA probes (vide infra), which will be discussed under the topic of PNA probes carrying fluorescent nucleobases (vide infra).

**Figure 10:**
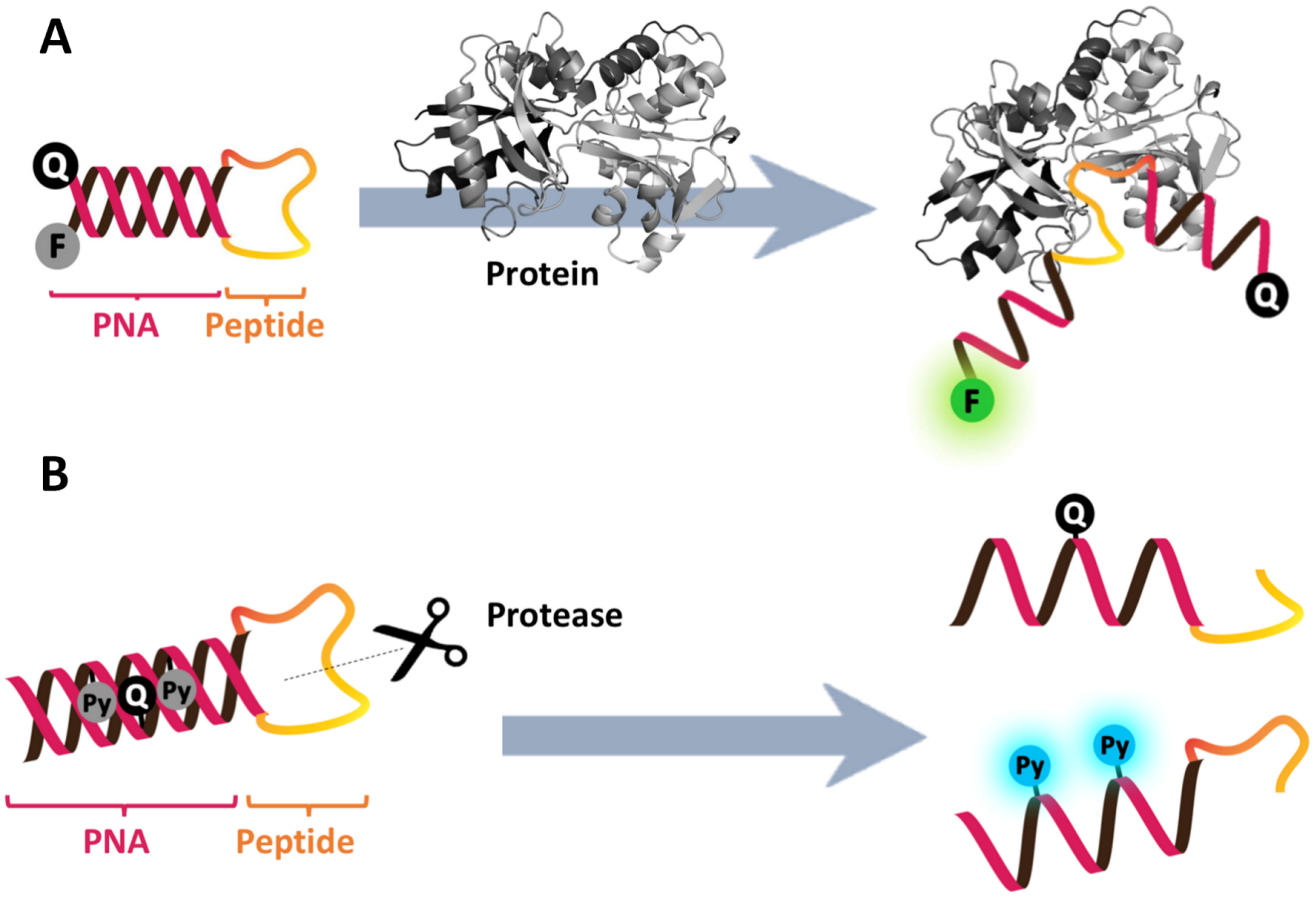
The working principle of hybrid PNA-peptide beacons for detection of (A) proteins [80] and (B) protease activities [82].

Dual-labeled PNA molecular beacons have been extensively used on their own or in combination with fluorescence melting curve analyses for genotyping [71–73] and analyses of genetic mutations, including single nucleotide polymorphisms (SNP), insertions and deletions [74–76]. They have also been used for the detection and quantification of ribosomal RNA in a wash-free FISH [77]. Side-by-side comparison showed that the PNA beacons gave faster hybridization kinetics, a higher signal-to-noise ratio and a much better specificity than DNA beacons. A PNA beacon has been used as a sensing probe for PCR amplicons in a droplet-based microfluidic device [78], while an integrated microfluidic device that combined sample preparation, hybridization and confocal fluorescence spectroscopy allowed amplification-free detection of 16S RNA from a single cell of *Escherichia coli* [79]. In addition to the detection of DNA and RNA targets, hybrid PNA-peptide beacons were designed for the detection of non-DNA targets, such as proteins (Figure 10A). According to this design, which was simultaneously proposed in 2007 by Seitz [80] and Plaxco [81], the peptide part acted as the recognition element while the labeled stem-forming PNA part acted as the switch. Binding of the target molecule resulted in a conformational change, leading to a change in the fluorescence signal. The same principle can be applied for the detection of protease activities (Figure 10B) [82]. The advantages of using PNA as the switch over DNA would be the excellent biological stability and the compatibility of PNA with peptide chemistry. Obviously, the ease of the opening of the beacon could be fine-tuned to accommodate the different strengths of the biomolecular interactions to be investigated by adjusting the length and sequence of the PNA stem.

#### PNA-based binary probes and DNA/RNA-templated reaction of PNA probes

**Figure 11:**
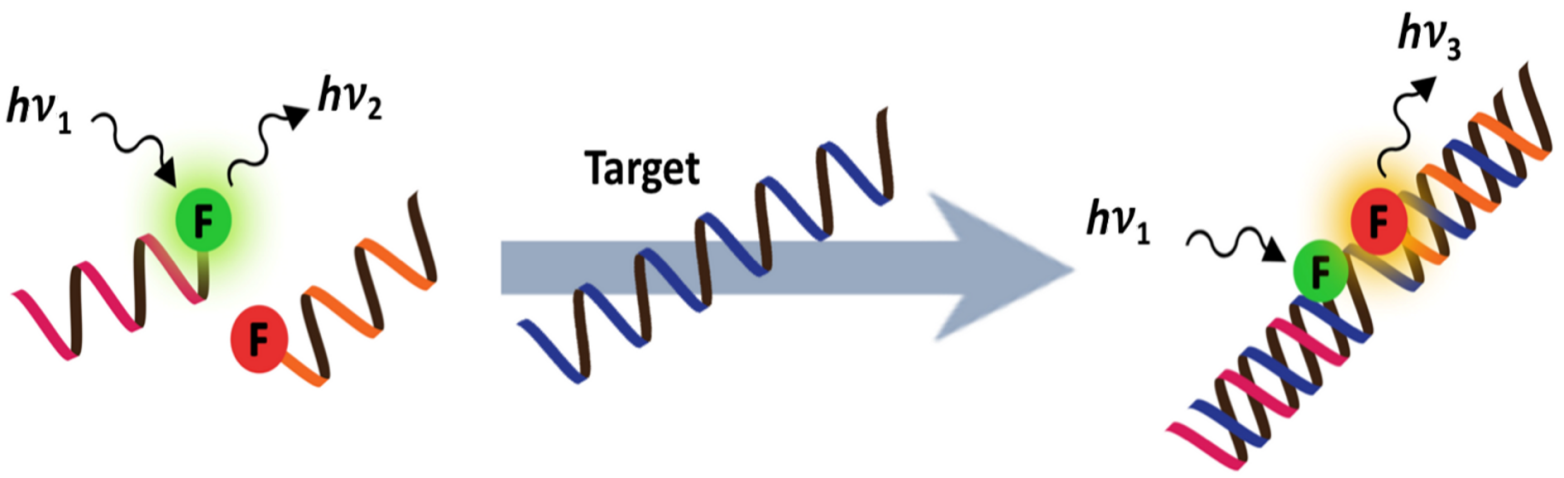
The working principle of binary probes.

The working principle of binary probes involves the co-hybridization of two dye-labeled probes at adjacent positions on the same nucleic acid scaffold or template, which allows the two dyes to optically interact, such as by FRET or excimer formation (Figure 11) [83]. In principle, the hybridization of two very short PNA probes should offer a better selectivity than a single longer PNA probe. The relative positions of the two labels, as well as the types of linkers, need to be fine-tuned in order to obtain optimal results. In this respect, the ability to site-specifically label anywhere in the PNA molecule using a pre-formed dye-labeled monomer or a functionalized monomer that allows post-synthetic labeling is important [84]. Accordingly, FRET-based binary PNA probes have been used to monitor RNA splicing whereby the decrease in distance between the two probes after splicing resulted in an increased FRET efficiency [85]. A fluorescent PNA probe was used for the selective amplification of a rare DNA mutation in K-ras by PCR clamping and, at the same time, as a sensor probe in combination with another dye-labeled DNA probe in a single-tube operation by real-time fluorescence monitoring [86]. More challenging applications of binary PNA probes for imaging mRNA expression in living cells is possible at a proof-of-concept stage, but there is still room for improvement [87].

**Figure 12:**
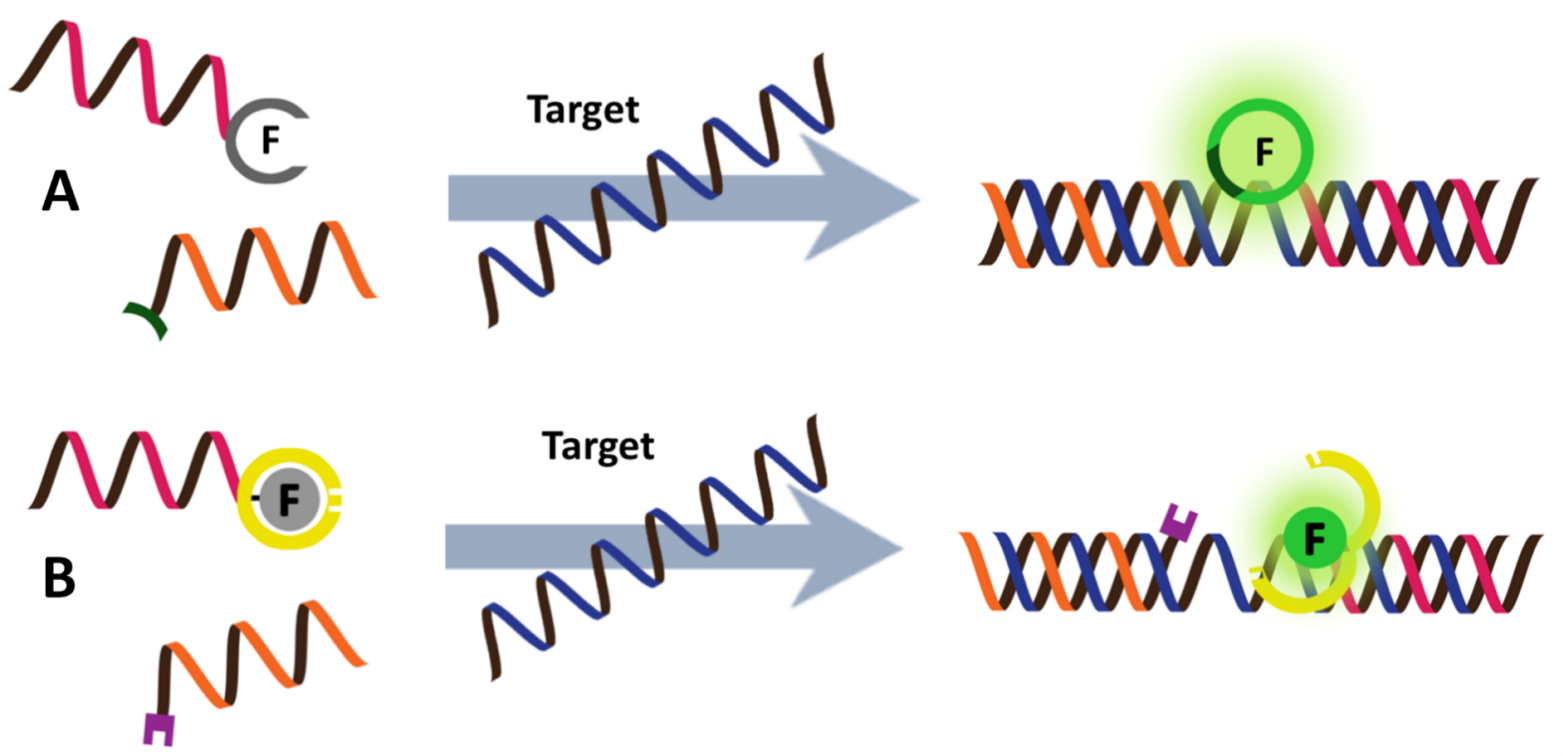
The working principle of nucleic acid templated fluorogenic reactions leading to a (A) ligated product and (B) non-ligated product.

**Figure 13:**
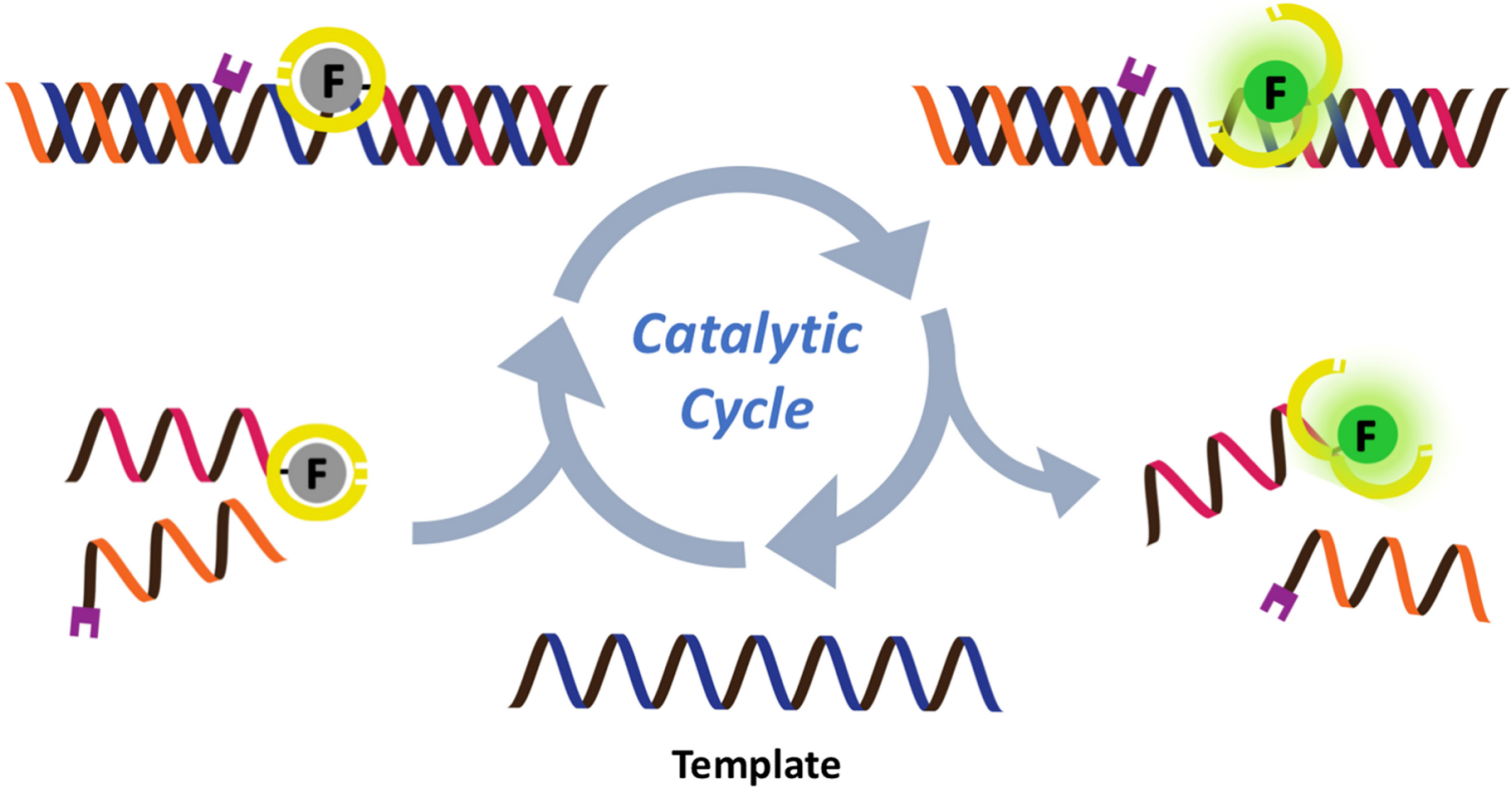
Catalytic cycles in fluorogenic nucleic acid templated reactions [90].

While numerous nucleic acid templated reactions involving oligonucleotide probes are known [88–89], only a few are PNA-based and fewer are fluorogenic. Each PNA probe carries one or more reactive groups that cannot react with each other at the low probe concentrations typically required for detection of the target molecules (nanomolar range or below). However, binding of the probes at adjacent positions on the same DNA or RNA template increases the local concentration of the probes so that they can readily react, resulting in creating and/or breaking one or more covalent bonds. They can be sub-divided into reactions that provide ligated products (Figure 12A) or non-ligated products (Figure 12B). In the latter case, the reacted probes readily dissociate and are replaced by unreacted probes, creating a catalytic cycle that leads to signal amplification (Figure 13) [90]. Signal amplification is also possible in the former case provided that the reaction was designed to give a ligated product that does not bind too tightly to the template, for example by using isocysteine-mediated native chemical ligation to provide a ligated PNA with a sub-optimal extended backbone at the ligation site [91].

Examples of the fluorogenic templated ligation of PNA are given in Table 1 which include the simple native chemical ligation of two fluorophore-labeled probes [92], formation of cyanine dyes [93–94], fluorogenic Michael addition [95–96] and cross-linking of dicysteine PNA probes that can bind with pro-fluorescent bisarsenical dyes [97]. Interestingly, in the native ligation reaction, the FRET efficiency increased substantially following the ligation even though the positions of the two PNA probes hardly changed [92]. It should also be noted that the selectivity for matched over mismatched templates for these short PNA probes (>100:1) is higher than in the case of molecular beacons or other DNA-based probes that are typically much longer [92].

**Table 1:** Examples of nucleic-acid-templated fluorogenic reactions of PNA probes leading to ligated products.

System	Remarks

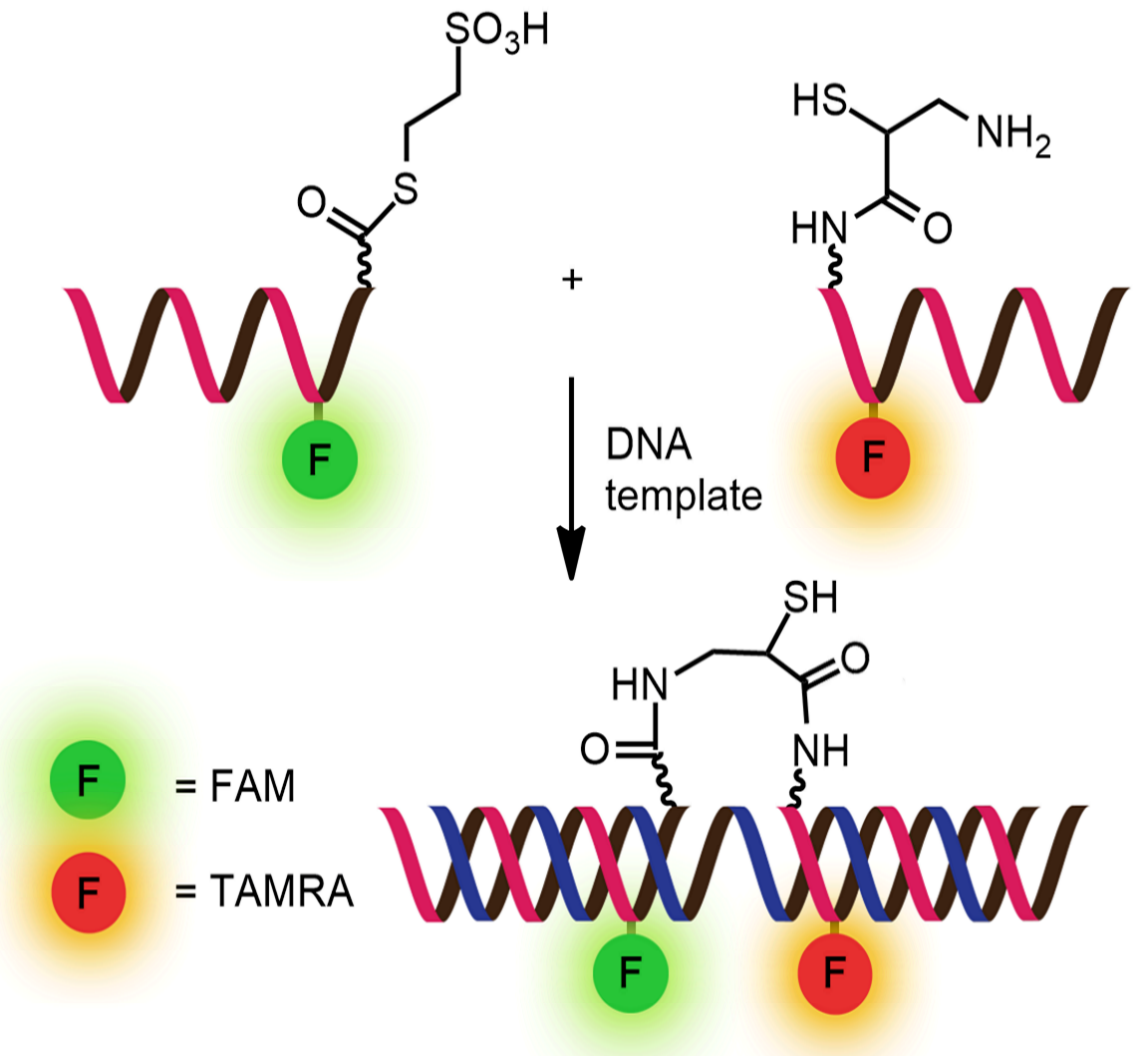	Reaction: native chemical ligation of two fluorescent labeled probes Probe: aegPNA Template: oligodeoxynucleotides Selectivity: 4.3 × 10^4^ (relative to non-templated reaction); 10^2^ to 10^3^ (relative to single-mismatched template) LOD: N/A (all experiments were conducted at 1 μM or higher concentrations) Applications: in vitro detection of oligodeoxynucleotides Ref: [92]
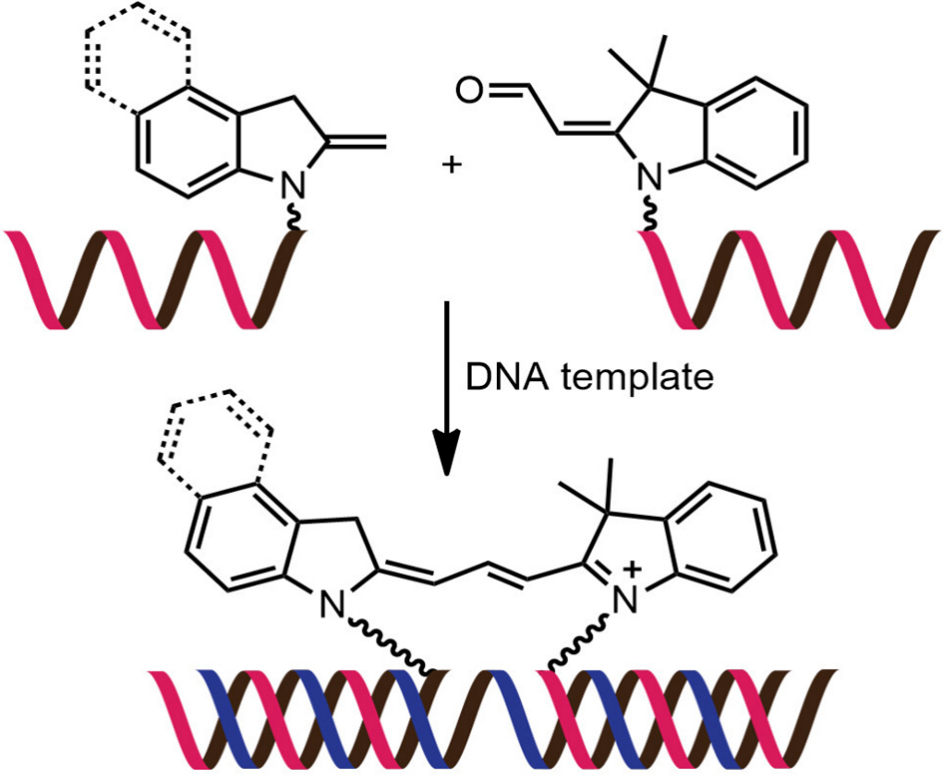	Reaction: formation of cyanine dyes Probe: aegPNA Template: oligodeoxynucleotides Selectivity: >50 (relative to non-templated reaction) LOD: <500 nM Applications: detection of conformational change of G-quadruplex- and hairpin-forming oligodeoxynucleotides Ref: [93–94]
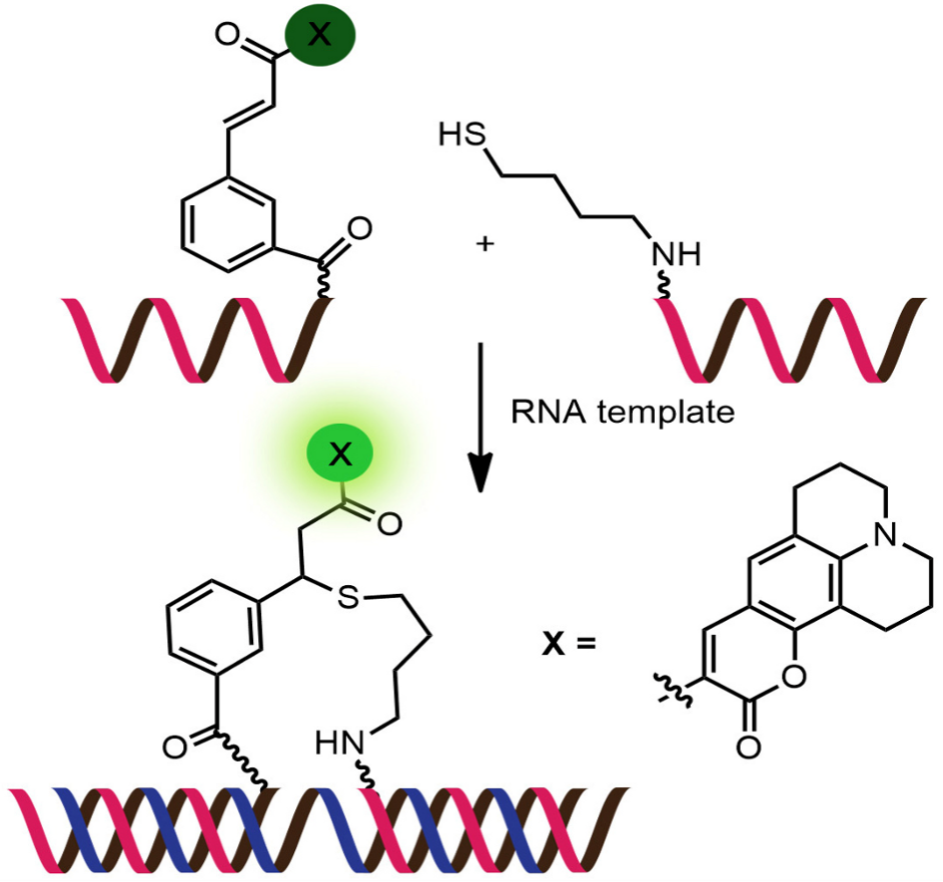	Reaction: Michael addition Probe: aegPNA Template: oligoribonucleotide Selectivity: ≈2–3 (relative to single-mismatched template); up to 80 (relative to non-templated reaction) LOD: ≈60 nM; improved to 0.1 nM by confinement in hydrogels Applications: amplification-free detection and quantitation of extracted miRNA biomarkers for prostate cancers (miRNA-141, miRNA-132, miRNA-375) Ref: [95–96]
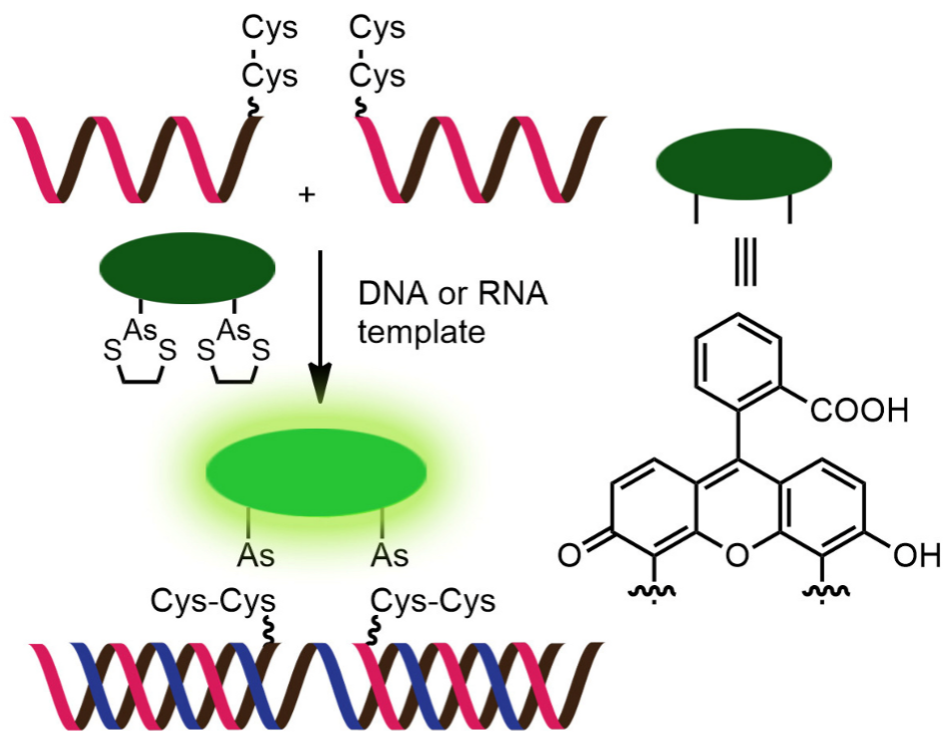	Reaction: cross-linking with bisarsenical dyes Probe: aegPNA Template: oligodeoxynucleotide and oligoribonucleotide Selectivity: ≈16 (relative to single-mismatched template); >50 (relative to non-templated reaction) LOD: 2.5 nM (without amplification); subnanomolar sensitivity for miRNA detection (with rolling circle amplification, RCA) Applications: in vitro detection of miRNA let-7a Ref: [97]

Examples of fluorogenic non-ligated templated reactions are summarized in Table 2. Many of these reactions feature the unmasking of quenched or caged fluorophores by chemical reactions, such as the Staudinger reaction [98–101], hydrolysis [102] and group transfer mediated by nucleophilic substitution [103–104]. Optimizing the conditions requires a good balance between the affinity of the probe to the template and the effective strand exchange. This can be achieved by performing the reaction at low probe concentrations and at a temperature close to the melting temperature of the probe-template complex [104]. Impressive performances of several of these systems have been demonstrated, mostly with synthetic oligonucleotide templates, and applications for intracellular nucleic acid detection are also emerging [90,100]. The unmasking by template-catalyzed hydrolysis or thiolysis is susceptible to other non-selective pathways, such as enzymatic hydrolysis, giving rise to false positive signals or a high background. The masking of fluorophores by a more stable group, such as azides, is more attractive. Unfortunately, the phosphine probe generally required for the unmasking of the azide probe is susceptible to rapid aerobic oxidation. Accordingly, new developments in this area are still required. One promising example is the release of fluorophores by a phosphine-free photocatalyzed reduction of a self-immolative azide-based linker [105].

**Table 2:** Examples of nucleic-acid-templated fluorogenic reactions of PNA probes leading to non-ligated products.

System	Remarks

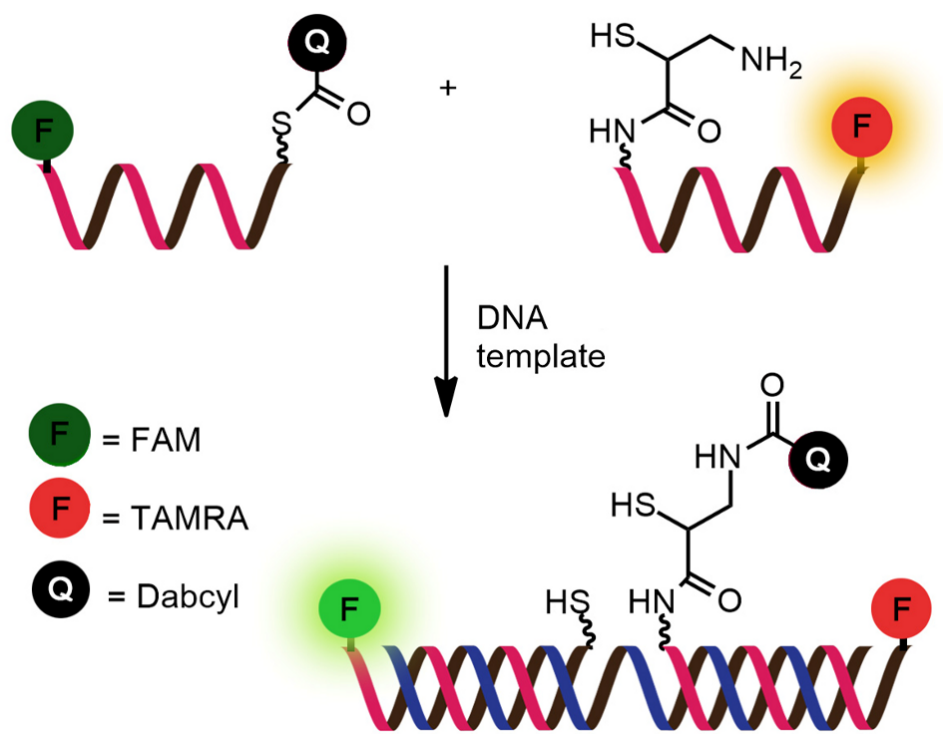	Reaction: thiol-mediated transfer of quencher Probe: aegPNA Template: oligodeoxynucleotide Rate acceleration: up to 1000 (relative to non-templated reaction) Selectivity: up to 138 (relative to single-mismatched template) TON: 8–38, depending on the type of linker LOD: 0.02 nM or less Applications: in vitro detection of oligodeoxynucleotides Ref: [103–104]
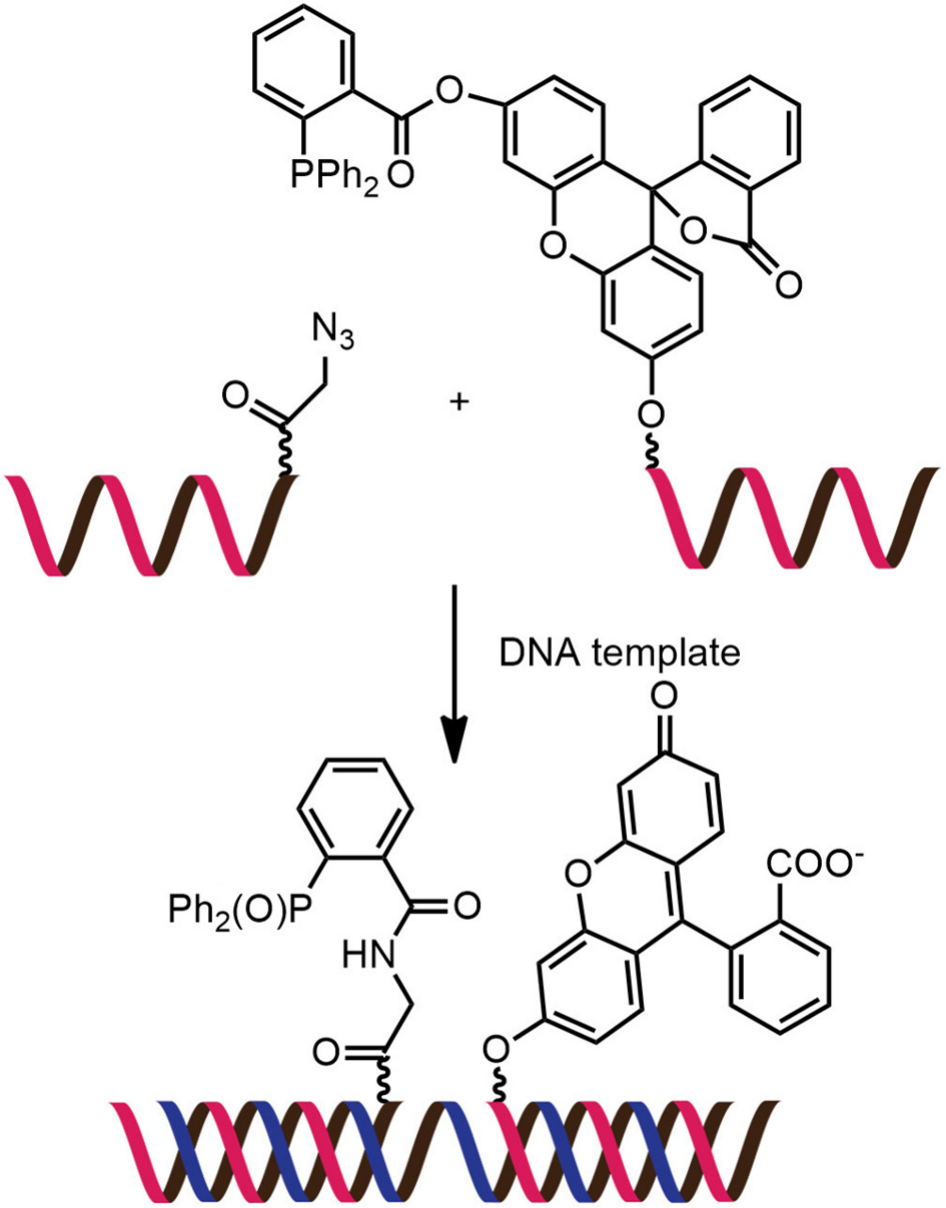	Reaction: uncaging of fluorophore by Staudinger reaction Probe: aegPNA Template: oligodeoxynucleotide Rate acceleration: ca. 188 (relative to non-templated reaction) Selectivity: 31–37 (relative to single-mismatched template) TON: N/A LOD: N/A (all experiments were conducted in low μM range) Applications: in vitro detection of oligodeoxynucleotides Ref: [98]
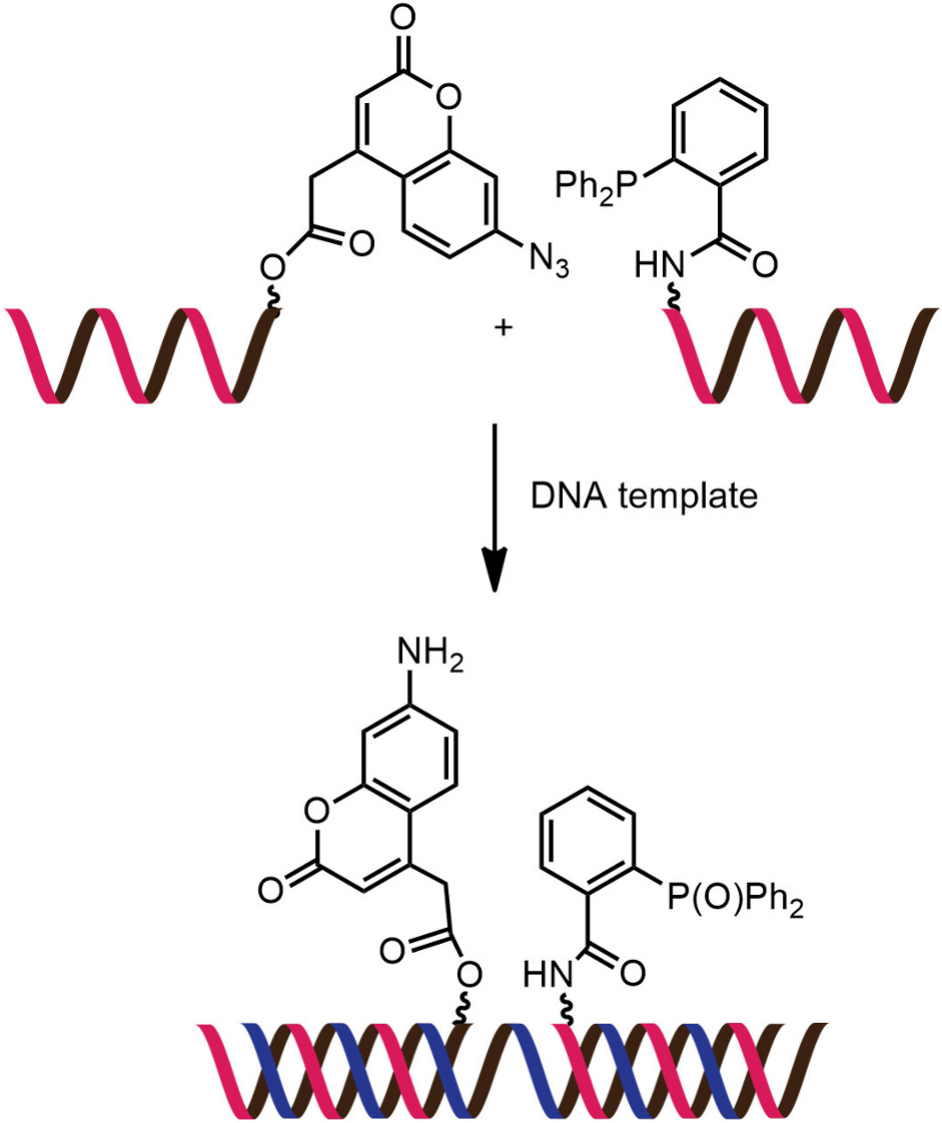	Reaction: uncaging of fluorophore by Staudinger reaction Probe: aegPNA Template: oligodeoxynucleotide Rate acceleration: N/A Selectivity: >10 (relative to single-mismatched template) TON: >100 LOD: low nM Applications: in vitro detection of oligodeoxynucleotides Ref: [99]
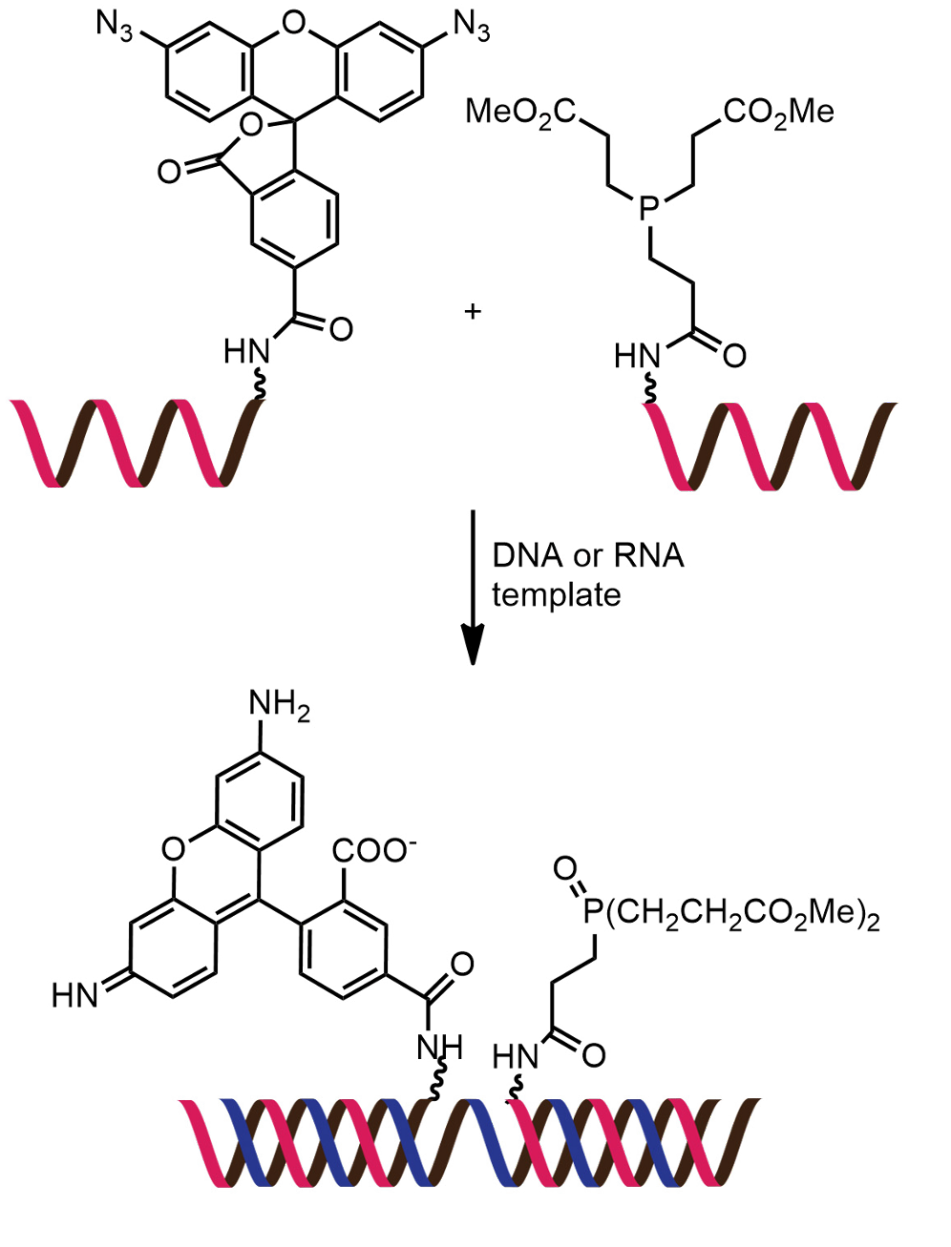	Reaction: uncaging of fluorophore by Staudinger reaction Probe: γGPNA Template: oligodeoxynucleotide and oligoribonucleotide Rate acceleration: N/A Selectivity: >10 (relative to single-mismatched template) TON: >10 LOD: low nM Applications: imaging of 23S RNA in fixed *E. coli* cells Ref: [101]
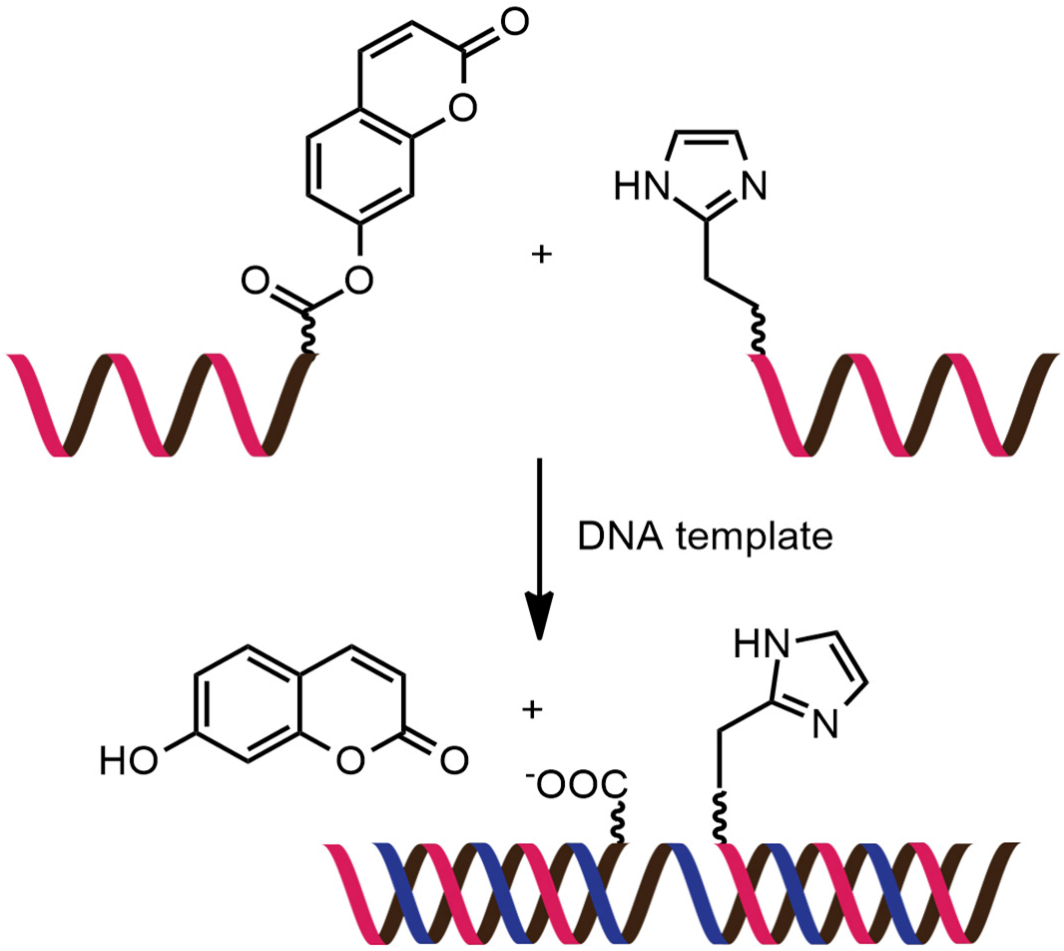	Reaction: unmasking of fluorophores by imidazole-catalyzed hydrolysis Probe: aegPNA Template: oligodeoxynucleotide Rate acceleration: 5.5 × 10^5^ (relative to non-templated reaction); 11 (relative to DNA-based reaction) Selectivity: 23–30 (relative to single-mismatched template) TON: N/A LOD: N/A (all experiments were conducted in low μM range) Applications: in vitro detection of oligodeoxynucleotides Ref: [102]
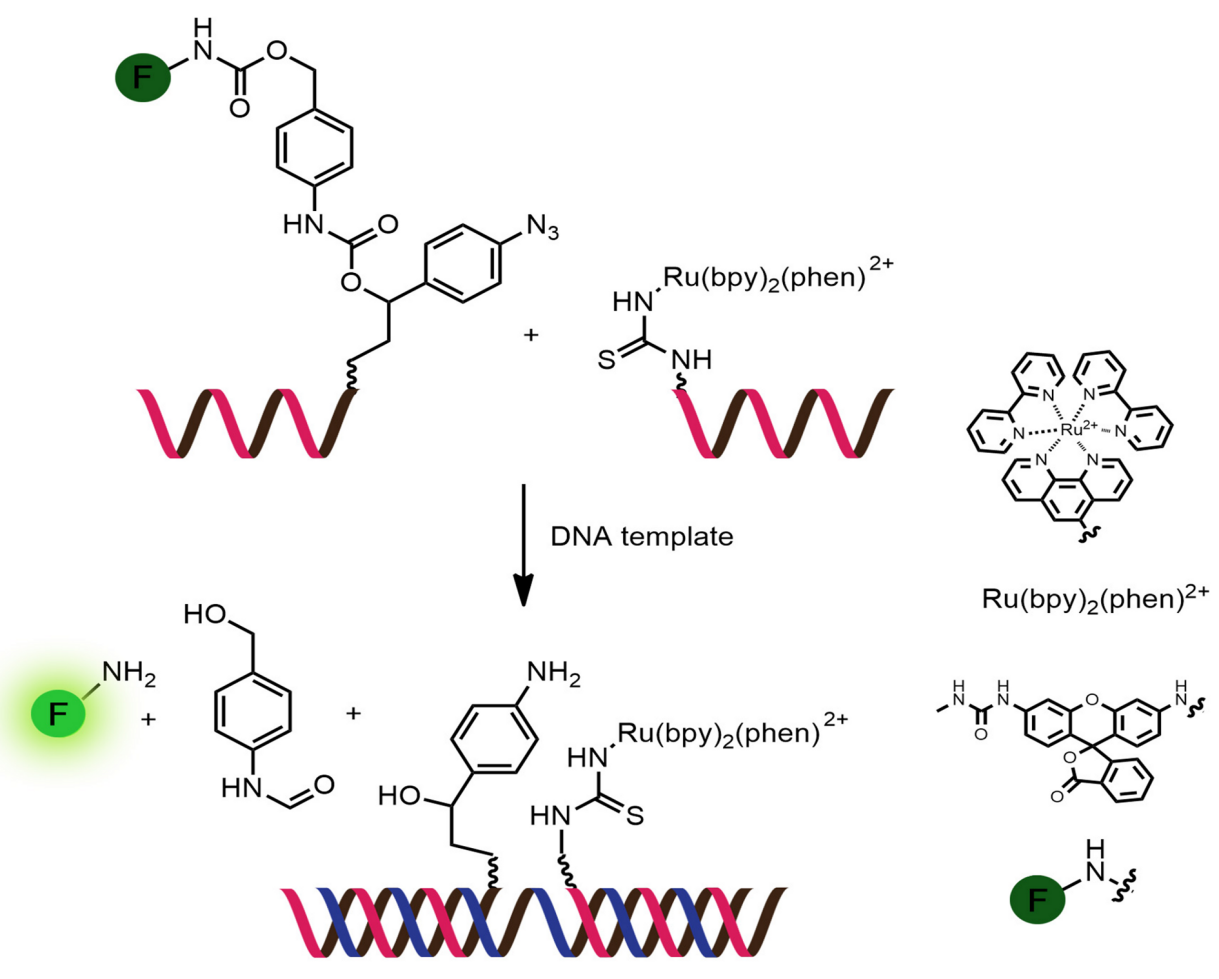	Reaction: photocatalytic release of fluorophore by reduction of self-immolative azide linker Probe: γ-Ser PNA Template: oligodeoxynucleotide, miRNA Rate acceleration: first order rate constant (*k*) 0.64 × 10^−3^ s^−1^; could not be measured for non-templated reaction Selectivity: 8–10 (relative to single-mismatched template) TON: >4000 LOD: 5 pM Applications: in vitro and intracellular visualization of miRNA Ref: [105]

### Single-labeled PNA probes with additional interacting partners

#### PNA-based strand displacement probes

**Figure 14:**
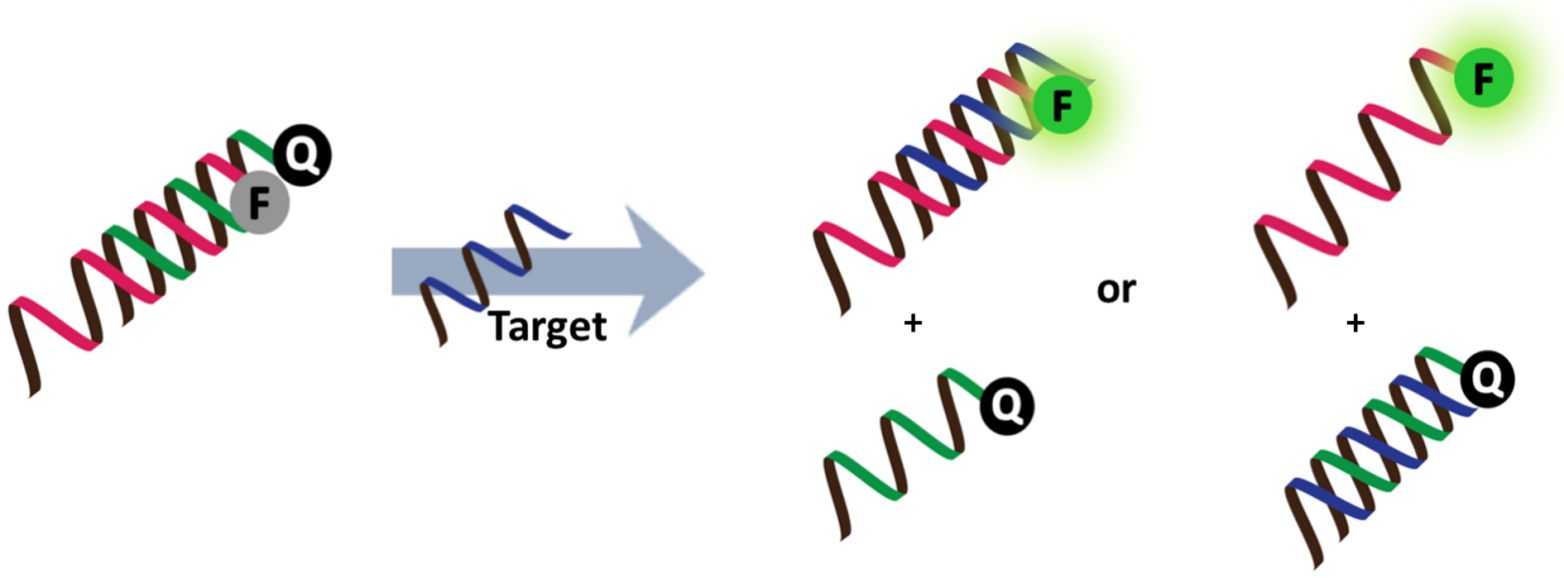
The working principle of strand displacement probes.

Strand displacement probes consist of a fluorescence oligonucleotide probe strand annealed to another oligonucleotide strand that is labeled with a second dye and which can interact with the first dye by quenching or FRET. In the presence of the complementary nucleic acid target, strand displacement takes place and results in separation of the two dyes, and so gives rise to a fluorescence change (Figure 14). The strand displacement probe combines the advantages of the design simplicity of linear probes with the high specificity of hairpin molecular beacons [49]. Labeled PNA has occasionally been used in combination with another DNA as a strand displacement probe. One of the earliest examples makes use of PNA probes immobilized on fluorescence-polymer-coated polystyrene microspheres and a quenching DNA strand [106]. The fluorescence of the microsphere was restored upon displacement of the quencher DNA strand by the DNA target in a sequence-dependent manner. Better mismatch discrimination was observed with PNA probes over DNA probes. In another example, a TAMRA-labeled PNA probe was used in combination with a short Cy5-labeled DNA oligonucleotide to form a FRET pair for the homogeneous assay of a SNP. Strand displacement with the unlabeled DNA target resulted in an increased TAMRA and decreased Cy5 fluorescence. Room temperature discrimination of a single base mismatch from the complementary DNA target was possible with a detection limit of 10 nM [107].

The use of pyrrolidinyl PNA probes gave an even better mismatch discrimination than conventional PNA probes [108–109]. The slow kinetics of the strand displacement involving highly stable PNA–DNA hybrids can be a major limitation of all the PNA-based strand displacement probes. Strand exchange can be facilitated by heating or increasing the salt concentration, where Mg^2+^ is more effective than Na^+^ [107]. On the other hand, a polycationic comb-type dextran-poly(lysine) copolymer was shown to strongly promote the strand exchange of PNA–DNA duplexes by another DNA strand [110]. The equilibrium of the strand displacement reaction lies on the side of the more stable duplex, and so the original strand displacement probes are designed to have lower stabilities than the final duplexes. This can generally be achieved by employing a short complementary strand or by introducing mismatch pairs into the probe [109], but a C·I pseudocomplementary base pair, which is somewhat less stable than the C·G pair, has also been successfully employed in one case [111]. A PNA-based strand displacement probe was delivered into mammalian cells by employing cationic shell cross-linked nanoparticles, whereupon its application in the imaging of cellular mRNA expression was demonstrated [112]. Related to the concept of strand displacement probes, a self-reporting PNA–DNA primer was designed, whereby a fluorophore-labeled DNA probe formed a duplex with a short quencher-labeled PNA strand. The sticky end of the DNA part in the chimeric probe acted as a primer in a PCR reaction, which, upon chain extension displaced the quencher PNA and resulted in an increased fluorescence after several rounds of PCR [113].

#### Combination of single-labeled PNA probes with cationic conjugated polymers

**Figure 15:**
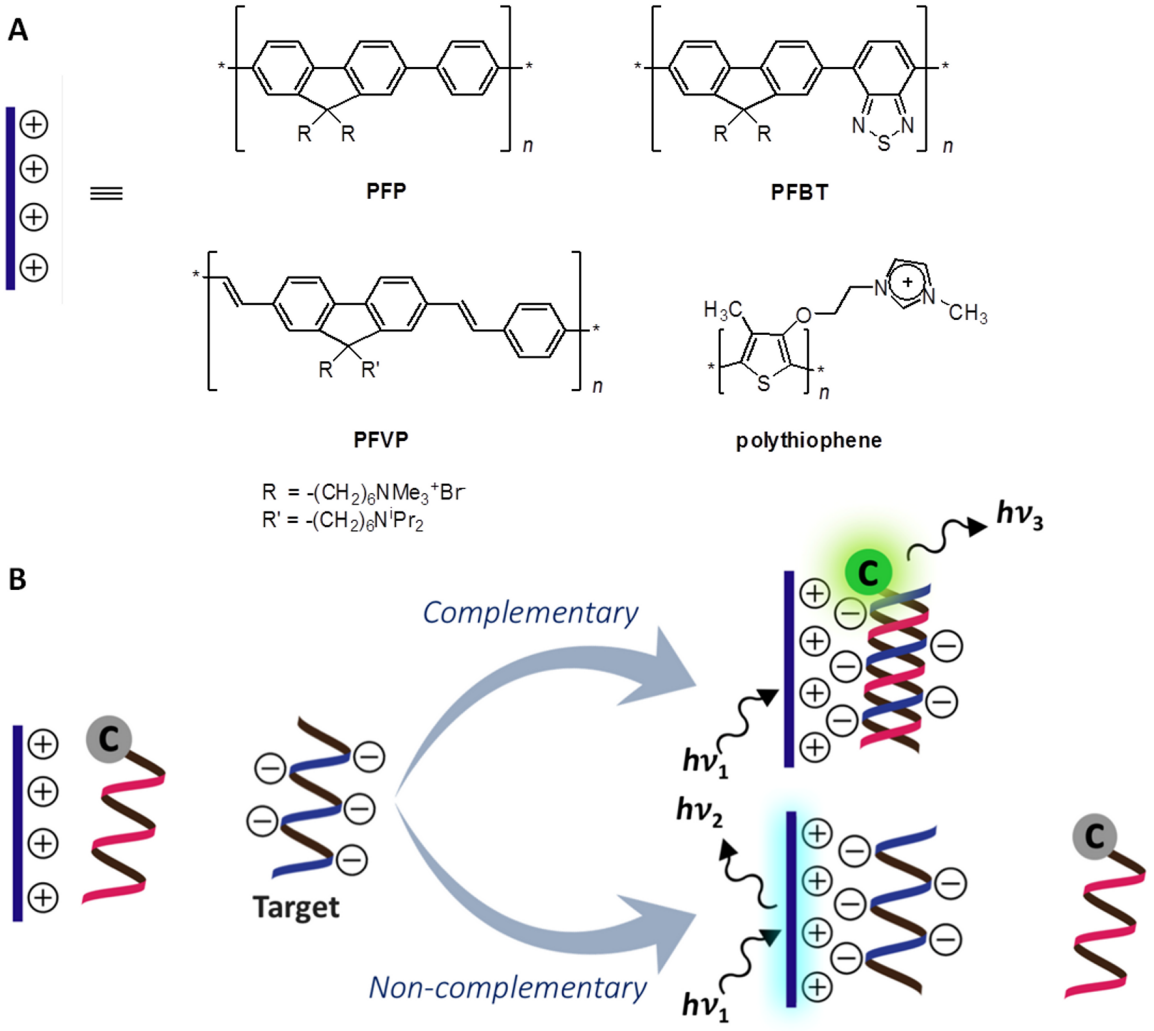
(A) Examples of CPP successfully used with labeled PNA probes. (B) The use of single-labeled PNA probes in combination with cationic conjugated polymers for FRET-based DNA detection.

The absence of negative charges on the PNA backbone offers a unique advantage for the development of novel DNA assays that cannot be made with oligonucleotides or analogues. One notable example is the use of a labeled PNA probe in combination with water-soluble cationic conjugated polyelectrolytes (CCP) for the FRET-based detection of DNA [114]. The CCP is typically an extended π-conjugated system, such as oligophenylene/fluorene with appending quaternary ammonium side chains (Figure 15A). Being electrostatically neutral, PNA cannot bind to these positively charged CCP unless it is first hybridized with DNA to form a negatively charged PNA–DNA complex. The CCP acts as a light harvesting antenna that transfers the energy to another label on the PNA probe and, therefore, the FRET is observed only when the labeled PNA probe, its complementary DNA and the CCP are present together (Figure 15B) [115–116]. It was demonstrated that the fluorescence signal obtained via FRET from CCP to fluorescein-labeled PNA was of an order of magnitude higher than the direct excitation of the fluorescein-labeled PNA [117]. Accordingly, the light harvesting properties of the oligomeric CCP improved the detection efficiency by signal amplification.

Although the high specificity of PNA probes can already differentiate between complementary and mismatched DNA targets on its own, the specificity for SNP detection could be further improved by the use of S1 nuclease, a single stranded specific nuclease that can digest single stranded DNA or mismatched duplexes more rapidly than perfect complementary duplexes [115]. The S1 digestion also improved the signal-to-background ratio by preventing the binding of the CCP on the DNA strand in the region remote from the PNA binding site, which is quite problematic in the detection of long DNA targets derived from PCR. On the other hand, addition of an organic solvent, such as *N*-methylpyrrolidinone [118], or a surfactant [119–120] improved the performance of the system by reducing the nonspecific aggregation of the hydrophobic CPP and by reducing its non-specific binding with ssDNA. Up to a 10-fold improvement in the detection sensitivity in the presence of sodium dodecylsulfate (SDS) was reported [121]. Detection of dsDNA targets via the formation of high order complexes between PNA–dsDNA was also possible using a combination of PNA probes and CPP [122]. The use of a labeled PNA probe can be avoided by employing a fluorescence dye that can bind to PNA–DNA duplexes or triplexes, such as thiazole orange (TO) [123]. A polycationic dendritic fluorophore has also been used as a FRET donor to fluorescein-labeled pyrrolidinyl PNA probes giving a highly sequence-specific DNA detection at room temperature without requiring S1 digestion at a subnanomolar detection limit [124].

A new CCP called PFBT that can be excited at 488 nm (the wavelength commonly found in commercial microarray readers) has been used in combination with Cy5-labeled PNA probes immobilized on glass slides for the FRET-based detection of DNA in microarray formats [125]. Approximately 10^10^ copies (ca. 20 fmol) of unlabeled DNA can be readily detected following standard surface-hybridization protocols. The use of the solid support assay format also allows detection of DNA directly from the fluorescence signal of the CPP bound to the solid support without requiring labeling on the PNA probe [126]. Polystyrene microbeads self-assembled on patterned silicon chips have been used as the solid support for the PNA–CPP based DNA assay. Using this assay format, a combination of long-wavelength emissive CPP and PNA allowed detection of as low as 150 attomol of unlabeled DNA, or 300 copies (0.5 zeptomol) of Cy5-labeled DNA targets by confocal microscopy [127]. A fluorescence cationic polythiophene that fluoresces upon binding to DNA [128] has been used as a transducer for the highly specific detection of DNA, captured by an unlabeled PNA probe immobilized on glass slides, giving a subpicomole sensitivity that could conceivably be improved by the use of labeled PNA probes [129].

#### Combination of PNA probes with nanomaterials as external quenchers/FRET partners

**Figure 16:**
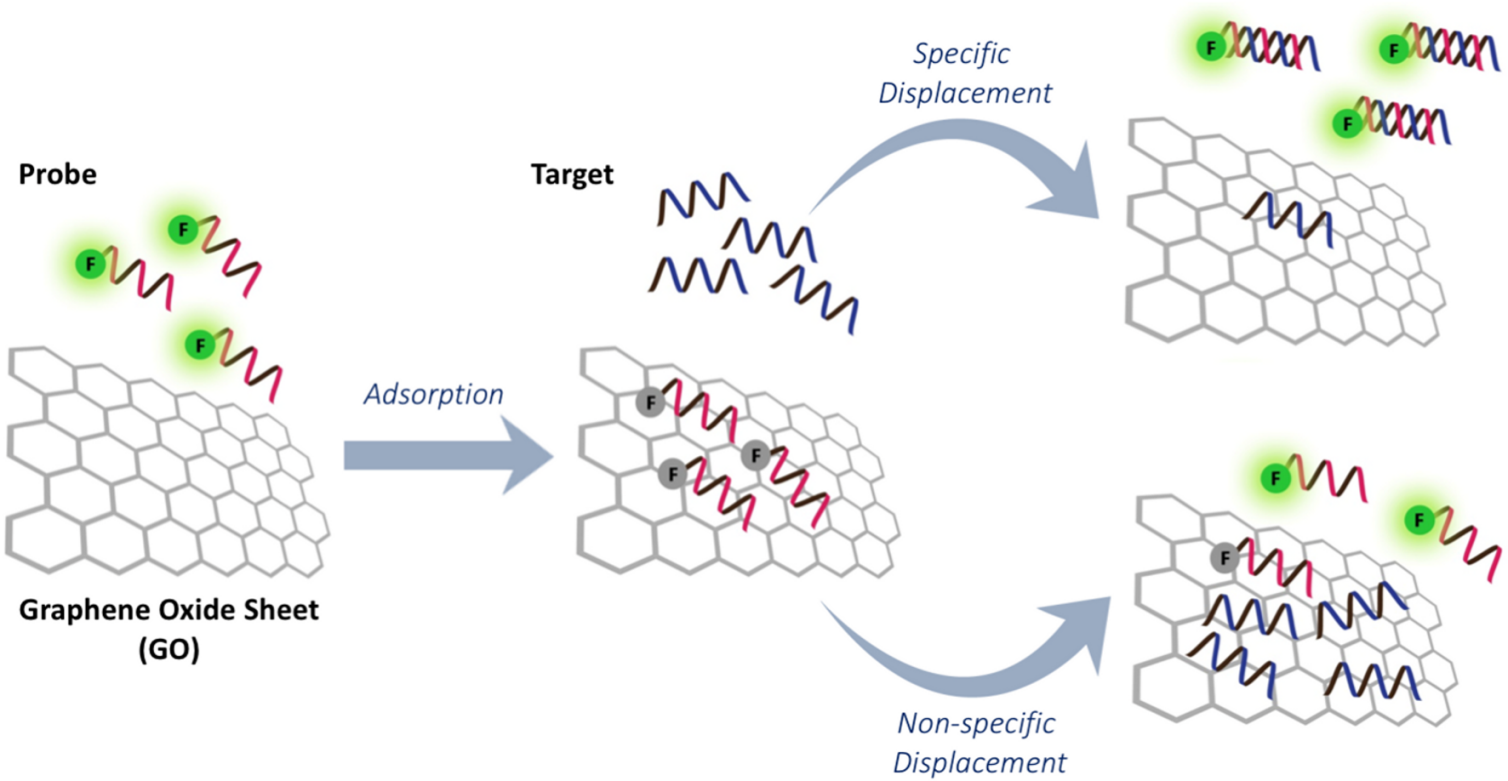
The concept of PNA–GO platform for DNA/RNA sensing.

Graphene oxide (GO) has been used extensively in combination with dye-labeled DNA for fluorescence-based DNA/RNA sensing [130–131]. Although ssDNA interacts strongly with GO via a combination of multiple interactions, it is mainly governed by hydrophobic interactions and hydrogen bonding between nucleobases and the GO surface [132]. Duplex DNA interacts less strongly with GO since the pairing nucleobases are buried inside the duplex in dsDNA. This phenomenon, together with the ability of GO to act as an effective fluorescence quencher, leads to the development of a novel fluorescence DNA sensor platform based on fluorescence labeled oligonucleotide probes and GO (Figure 16) [133–134]. Although the applications of DNA-based fluorescent probes have been well documented, the non-specific nature of the interaction between ssDNA and GO can result in competitive displacement of the adsorbed DNA probes by non-target ssDNA [135–137]. The degree of such non-specific displacement would depend strongly on the base sequence and, therefore, false positive results can be expected.

The use of PNA as a probe offers a solution to overcome this issue since the hydrophobic backbone of the PNA probe appears to enhance the interaction with GO further, making the non-specific displacement of PNA by non-target DNA unfavorable [138]. The stronger interaction also means that fluorescent labeled PNA probes are more strongly quenched by GO than DNA probes with an identical sequence, leading to a lower background fluorescence. Importantly, the adsorbed PNA probes are still available for the formation of PNA–DNA duplexes, which are less strongly adsorbed by GO and so this leads to a release of the hybridized PNA probe from the GO and an enhanced fluorescence. The drawback of the use of the strongly adsorbed PNA probes is the rather slow kinetics and therefore an elevated temperature is often required to promote the desorption and hybridization of the PNA probes. Detection limits in the nano- to picomolar range have been achieved, while the high specificity of PNA allowed differentiation between complementary and non-complementary DNA, including single mismatched DNA as well as RNA targets, although temperature shifting is often necessary to obtain perfect discrimination [139–140].

The ability of PNA to invade into the DNA duplex allows the use of a PNA–GO sensing platform for the direct detection of dsDNA targets without requiring denaturation. Using three different labeled PNA probes, multiplex detection of short synthetic dsDNA that corresponded to the DNA sequences of HVA (hepatitis A Val17 polyprotein), HIV and HVB (hepatitis B virus surface antigen) in a single tube was demonstrated [141]. Addition of a very low concentration of bovine serum albumin (BSA, 0.01%) was shown to enhance the performance of the PNA–GO-based DNA sensing platform by reducing the non-specific adsorption of PNA–DNA duplexes to GO, which resulted in a lower limit of detection (LOD) by almost two orders of magnitude [142]. The detection limit could be improved further for the detection of microRNA by a sequence specific RNA-templated DNA ligation to form a circular DNA. This circular DNA was then used as a template for rolling circle amplification reaction (RCA) initiated by phi29 DNA polymerase to give a long tandem repeat DNA. As low as 0.4 pM of miRNA could be readily detected with single mismatch specificity [143].

In addition to the direct detection of DNA and RNA targets, the PNA–GO platform has also been used for an in vitro assay of RNA polymerase activities and its inhibition by detection of the RNA formed [144]. The cell-penetrating ability of nanosized GO (NGO) allowed cellular internalization of the PNA–NGO complexes, enabling direct, quantitative and multiplex monitoring of various micro (mi)RNAs in living cells [145]. Side-by-side comparison showed clear advantages of PNA over DNA probes, where the DNA–NGO complexes lighted up non-specifically in the presence of the cell lysate, presumably by the interference of cellular matrices with the DNA–NGO interactions or by cleavage of the DNA probes by cellular nucleases. Related to this, hyarulonic acid-coated GO was also employed as a carrier for introducing PNA into cancer cells for the simultaneous detection and inhibition of endogeneous miRNA-21 in CD44-positive MBA-MB231 cells, leading to a decreased proliferation and inducing apoptosis [146].

In addition to GO, several other nanomaterials have been used as external quenchers in combination with labeled PNA probes for the fluorescent detection of DNA or RNA. WS_2_ nanosheets can selectively adsorb ssPNA and quench its fluorescence [147]. The addition of complementary DNA targets resulted in the restoration of the fluorescence in pretty much the same way as GO. Likewise, carbon nitride nanosheets [148] and a zirconium-based nano metal-organic framework (UiO-66) [149] behaved similarly. In addition, they have been successfully used in combination with PNA for monitoring of miRNA inside living cells [148–149].

Quantum dots (QDs) are another class of nanomaterial that offer great promise as a FRET partner for fluorescence detection of nucleic acids [150–153]. Despite the fact that several examples of QDs and oligonucleotide probes for DNA/RNA detection are known [154–155], the use of QDs in combination with labeled PNA probes was only recently reported for a sandwich-type fluorescence detection of DNA at nanomolar concentrations [156].

### Single-labeled fluorogenic PNA probes

**Figure 17:**
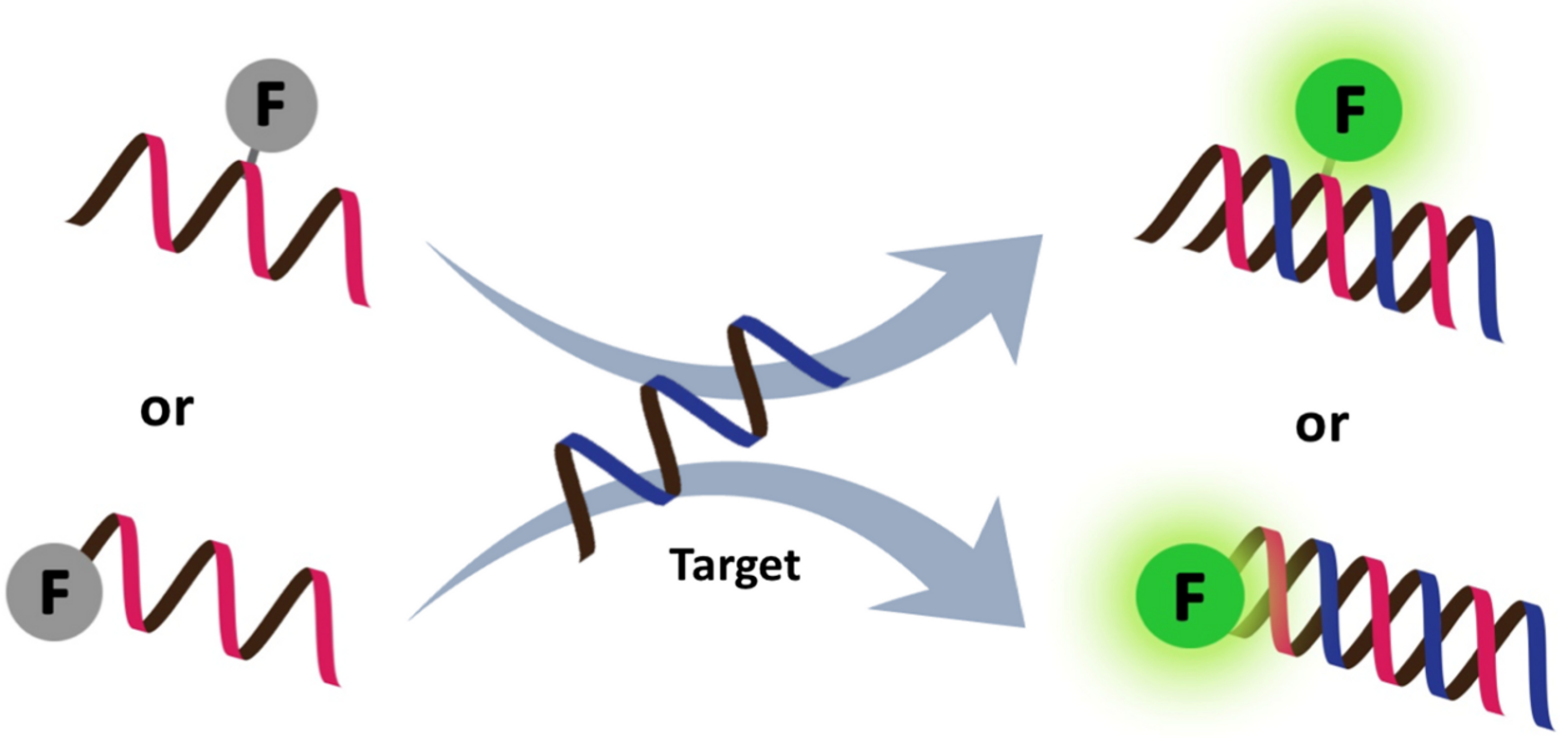
Single-labeled fluorogenic PNA probes.

Fluorogenic linear PNA probes carrying a single label (Figure 17) are highly attractive in terms of their ease of design and synthesis. To allow the wash-free, homogeneous detection of target nucleic acids, it is necessary to incorporate an environment sensitive label onto the PNA probe. Ideally, the label should interact differently and yield different responses to ssDNA, ssPNA and PNA–DNA or PNA–RNA duplexes by various mechanisms, such as groove binding or intercalation. This fluorescent label may be a stand-alone entity (referred to as a tethered label) or combined with a nucleobase that integrates the base pairing and the fluorescence sensing in a single event. The latter strategy should allow a more precise control of the position and orientation of the label than the former, and therefore should be more sensitive to local environment changes than the former, which indirectly and non-specifically senses the global formation of duplex or base stack.

#### PNA probes with tethered labels

**Figure 18:**
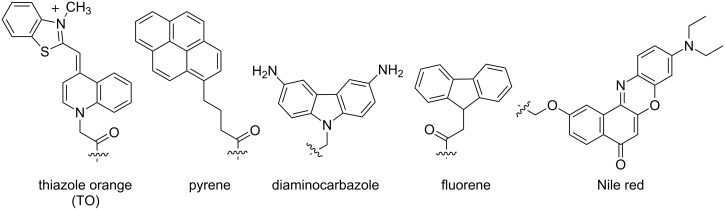
Examples of environment sensitive fluorescent labels that have been incorporated into PNA probes as a tethered label.

**Figure 19:**
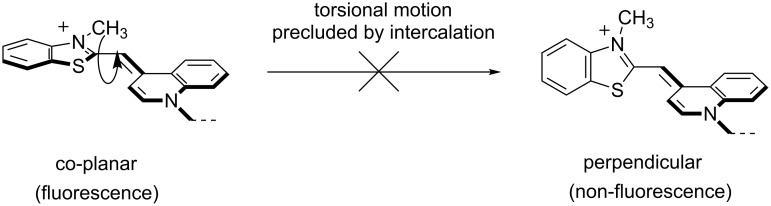
The mechanism of fluorescence change in TO dye.

One of the earliest PNA probes in this category are the so-called "light-up probes", which consist of a short single stranded PNA probe (ssPNA) linked to the DNA binding dye TO (Figure 18) at one end of the molecule, usually at the N-terminus, via a flexible linker [157]. The TO binds non-specifically with DNA duplexes and shows a large fluorescence enhancement upon binding [158]. PNA–DNA hybridization facilitates the binding of TO to the PNA–DNA duplexes, resulting in a fluorescence enhancement due to the increased co-planarity and restricted motion of the two conjugated aromatic rings in the TO (Figure 19). The electrostatically neutral backbone of PNA was originally thought to offer a unique advantage over DNA and other negatively charged oligonucleotide analogues, since it would minimize self-binding between TO and the probe itself. However, subsequent studies have revealed that there are still significant fractions of TO that are back-bound to the PNA probe (molar ratio between 0.7 to almost 1.0 at 30 °C) [159]. The smallest fraction was observed in probes with homopyrimidine sequences. In addition, the fluorescence quantum yields of the probe in the back-bound conformation were lowest in pyrimidine rich sequences. Therefore, the performance of light-up probes is rather sequence-dependent, although it could be marginally improved by increasing the temperature.

Rapid and specific detection of PCR products by light-up PNA probes in a simple mix-and-read fluorescence assay [160] or by real-time PCR [161–162] have been demonstrated in the context of pyrimidine rich sequences. A pair of pseudo-complementary PNA probes terminally labeled with TO allowed the detection of specific DNA sequences in long dsDNA and plasmids without requiring prior denaturation [163]. Although non-selective binding of the TO label to DNA or RNA duplexes were occasionally observed, this could be suppressed by using a shorter linker (as in FIT PNA probes, vide infra) or by combination with a second dye, such as pyrene, that can interact with TO by π-stacking. Such a combination in a short PNA probe has been used as a non-covalent affinity label for monitoring small interfering (si)RNA delivery [164–165]. In addition, TO has also been linked as a tethered label at internal positions of γPNA [166] and acpcPNA [167], where both showed dramatic light-up behaviors (50–100 fold) when hybridized to complementary DNA.

In addition to TO, other environment-sensitive labels, such as fluorene [168], pyrene [167,169–170], diaminocarbazole [171] and Nile red [172], have been successfully employed in the development of single-labeled fluorogenic aegPNA, γPNA and acpcPNA probes (Figure 18). Up to a 73-fold fluorescence enhancement was observed in the case of a single pyrene-labeled acpcPNA probe [170]. This is in sharp contrast to pyrene-tethered DNA probes, which are usually quenched after hybridization due to intercalation of the pyrene in the DNA–DNA duplex [173–174], unless the pyrene is attached to the base via a rigid linker that disfavors such intercalation [175–176].

#### PNA probes carrying fluorescent nucleobases

There are three major strategies to introduce fluorescent nucleobases into oligonucleotides or PNA probes. These are (i) the extension of conjugation in the natural nucleobase, (ii) appending a fluorophore onto a natural nucleobase and (iii) the use of unnatural, intrinsically fluorescent nucleobases. They can be divided into fluorescent nucleobases capable of hydrogen bond formation, which can form specific base pairs with canonical nucleobases, and those that cannot (i.e., universal bases). In this section, only those that have been used in combination with PNA will be discussed. More general reviews of fluorescent base analogues in the DNA/RNA context can be found elsewhere [177–178].

#### Fluorescent nucleobases capable of hydrogen bonding ("base discriminating fluorophores")

**Figure 20:**
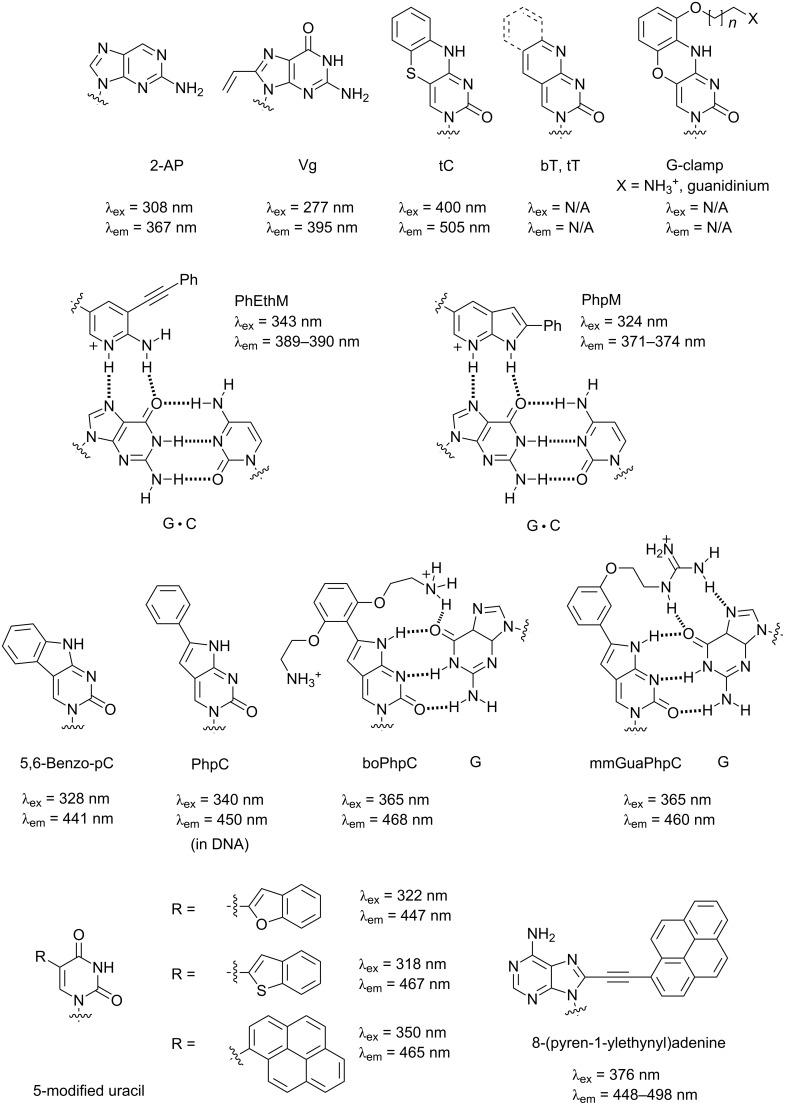
Fluorescent nucleobases capable of hydrogen bonding that have been incorporated into PNA probes.

The structures of fluorescent nucleobases with hydrogen-bonding abilities that have been studied in the PNA context are shown in Figure 20. The earliest examples of PNA carrying an intrinsically fluorescent nucleobase 2-aminopurine (2-AP) were reported since 1997 [179]. The 2-AP in aegPNA selectively recognizes dT in the DNA strand with comparable affinity to that for A, and the base pairing resulted in fluorescence quenching. While the quenching of 2-AP can be useful for probing the structure and dynamics of the PNA and its DNA/RNA hybrids, the quenching effect is small and not highly specific as quenching may also be partly observed in the case of mis-pairing [180]. In another example, the fluorescence 8-vinylguanine (Vg) in aegPNA forms specific base pairs with C in DNA and RNA. In addition, Vg can still participate in G-quadruplex formation similar to dG. The formation of base pairs or G-quadruplexes was accompanied by fluorescence quenching, and Vg-modified aegPNA has been used to probe the interaction between RNA quadruplexes and PNA [181].

Another example of a nucleobase with extended conjugation that has been incorporated into PNA is 1,3-diaza-2-oxophenothiazine (tC), which is a tricyclic analogue of cytosine [182]. The tC exhibits decent quantum yields of ≈0.2 both in free form and when incorporated into DNA or aegPNA [183], and forms specific base pairs with dG, but does not show appreciable fluorescence change upon the base pairing. While this could be advantageous for several applications, it means that tC–PNA alone is not useful as a fluorogenic probe. The related tri- or bicyclic thymine analogues tT and bT (without the additional aromatic ring) pair specifically with dA when incorporated into aegPNA, but their fluorescence properties have not been reported [184]. PNA carrying phenoxazine analogues of tC (tC^O^) with a positively-charged pendant (so-called "G-clamp") were prepared in order to improve the affinity of PNA–DNA duplex by additional hydrogen bonding [185–187]. No fluorescence properties of these tC^O^-based G-clamps PNA have been reported, but it is quite likely that they will be non-responsive to the base pairing similar to PNA carrying tC or tC^O^ [177].

Pyrrolocytosine is another intrinsically fluorescent hydrogen-bond-forming nucleobase that has been extensively studied in a DNA context [188–189]. When incorporated into PNA, the simple phenyl-substituted pyrrolocytosine (PhpC) recognizes dG in DNA and G in RNA with a slightly increased and decreased affinity, respectively, relative to C [190]. Addition of a positively charged pendant group at the *ortho*-position of the phenyl substituent, such as in boPhpC, substantially increased the binding affinity as well as the specificity due to the additional hydrogen-bonding interactions with the pairing G residue analogous to G-clamp phenoxazine. Moving the substituent to the *meta*-position, as in mmGuaPhpC, increased the binding affinity towards RNA while still maintaining good DNA binding [191]. When incorporated into PNA, these PhpC derivatives exhibited a bright blue fluorescence with large fluorescence quantum yield (0.5–0.6), which can be useful for monitoring the cellular uptake and distribution of PNA [192]. In addition, the fluorescence was responsive to the base pairing, where up to 60% quenching was observed upon duplex formation with DNA or RNA. The fused ring fluorescence cytosine analogue 5,6-benzo-pC gave a large Stokes shift (113 nm) and good quantum yield (0.79) as a monomer. Unfortunately, severe fluorescence quenching was observed upon incorporation of this monomer into PNA sequences and no discrimination was observed among complementary and mismatched DNA targets [193]. PNA probes carrying PhpM, a deoxy analogue of PhpC, and its open-chain analogue PhEthM were designed as a fluorogenic probe for the detection of RNA duplexes. Isothermal calorimetric titration suggested that PhpM was less selective than PhEthM in recognizing dsRNA, and that the binding was pH dependent. A lower pH was required for protonation of the nitrogen atom to allow binding with G·C base pairs in a Hoogsteen fashion. Moderate fluorescence quenching (50–60%) was observed upon triplex formation [194].

Fluorophore-modified uracils have also been extensively studied as fluorescent nucleobases in the context of aegPNA. Modifications have invariably been made at the 5-position of uracil, which could be functionalized by various aromatics or alkynes via palladium-catalyzed cross couplings of the corresponding iodouridine derivative. 5-Benzothiophene- and 5-benzofuran-modified uracil in aegPNA exhibited a fluorescence that was marginally sensitive to the environment [195]. When incorporated into PNA, benzothiophene-uracil exhibited an increased fluorescence compared to the free nucleoside. The opposite effect was observed with benzofuran-uracil, and the fluorescence was almost completely quenched when G was the flanking nucleobase. Significant fluorescence enhancement with a small blue-shift of the emission maxima was observed upon duplex formation with DNA for both modified uracil derivatives. Unfortunately, the discrimination between complementary and mismatched duplex observed with benzothiophene-uracil was limited to sequences with flanking Cs. No discrimination was observed with benzofuran-uracil PNA unless it was used in combination with GO as a quencher for ssPNA [196]. In this respect, the thiophene-modified uracil acted rather like a general fluorescence label.

On the other hand, the fluorescent nucleobase 5-(pyren-1-yl)uracil in acpcPNA formed a specific Watson–Crick type base pairing with dA in the DNA strand, and the duplex formation was accompanied by a strong (up to 42-fold) fluorescence emission increase at 465 nm [197]. This is in sharp contrast with the behavior of the same pyrene-modified uracil in DNA, where no discrimination was observed among the four canonical nucleobases in terms of both thermal stabilities and fluorescence responses [198–199].

A similar selective recognition of dT with light-up behavior was also observed with the fluorescent nucleobase 9-(pyrenylethynyl)adenine when incorporated in acpcPNA, but not in DNA [200]. In the case of DNA–DNA duplexes, the nucleobase most likely adopts a *syn* conformation, thereby placing the hydrophobic pyrene moiety in the base stack at the expense of hydrogen bonding. The stronger base-pairing in PNA–DNA duplexes probably make this process less favorable. This emphasizes the subtle different behavior of PNA and DNA that may make PNA useful in certain circumstances.

#### Fluorescent nucleobases incapable of hydrogen bonding

**Figure 21:**
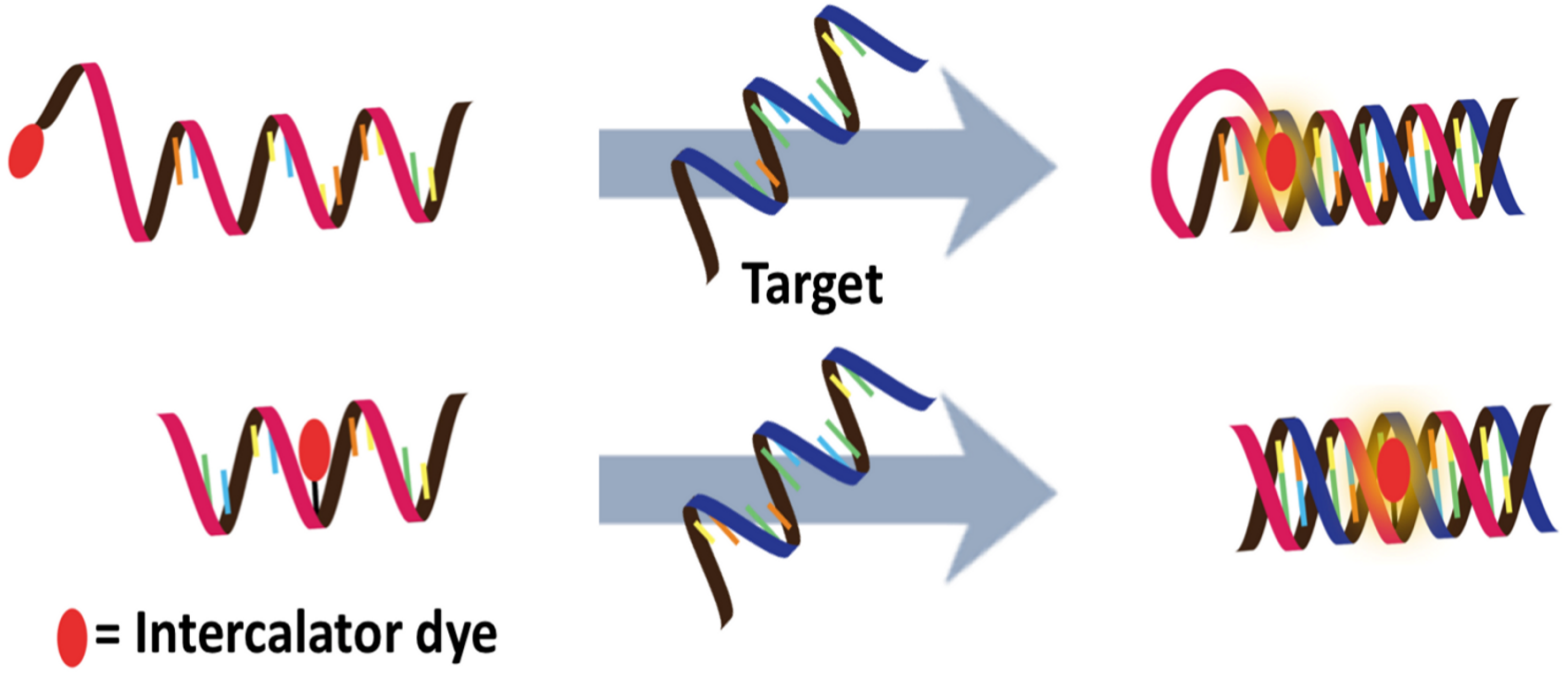
Comparison of the designs of the (A) light-up PNA probe and (B) FIT PNA probe.

The FIT probe first reported in 1999 [201] is perhaps the most well-known representative PNA probe in this class [202–203]. It contains the DNA-staining dye TO attached to the backbone of aegPNA as a nucleobase surrogate (Figure 21). Being incapable of hydrogen binding, TO behaves as a universal nucleobase as it can pair equally well with all canonical nucleobases in the DNA strand [204]. However, unlike most universal bases that usually destabilize the duplex [205], the base pairing strength involving TO was comparable to that of A·T pairs. The fluorescence of the TO-based FIT probe was increased substantially in the presence of the complementary DNA target as a result of the restricted rotation of the TO chromophore upon its intercalation into the base stack (Figure 19). Considering the bulkiness of the TO dye, it is quite likely that the opposite nucleobase was forced away from the duplex and so did not directly participate in the base pairing. The responsiveness of TO is sequence-dependent, and the largest responses (>20-fold) were observed when there is at least one A residue adjacent to the TO base. In most cases the fluorescence increase is quite general for various sequence context [206], Nevertheless, a smaller fluorescence changes (less than 10-fold) were observed with other flanking nucleobases. Importantly, when a mismatched base pair is present adjacent to the TO, the fluorescence increase was much lower than the complementary duplex, and the discrimination could be improved further, albeit at the expense of sensitivity, by increasing the temperature.

Unlike light-up probes, whereby the TO was linked at the end of the PNA probe as a tethered label via a long and flexible linker, FIT probes have the TO linked to the PNA backbone as a base replacement via a short and rigid linker (Figure 21). The working principle of the FIT probe is, therefore, distinctly different from light-up probes and should in principle allow for better control since the TO is forced to intercalate into the duplex at a well-defined position and so can directly sense the mismatched base pairing adjacent to the intercalation site. In contrast, the tethered TO label in a light-up probe globally senses the duplex formation by intercalation of the dye into the PNA–DNA duplexes making it difficult to differentiate between the complementary and mismatched duplexes. The residual fluorescence of the single stranded FIT probe was also more easily predicted than that of light-up probes since it is the result of direct interaction between the TO and the nearest neighbor, and an adjacent G is to be avoided since it contributes to a large background signal [206].

Detailed studies of the fluorescence lifetime indicated several distinct fluorescence decay processes are associated with different hybridization states of FIT probes [207]. Systematic variation of the linker length and position of attachment of the TO label indicated that the best response and mismatch discrimination were observed with a short linker linked to the quinoline ring of the TO [208]. Interestingly, a larger fluorescence increase was observed in the mismatched than complementary duplex when the TO dye was linked to PNA via the benzothiazole ring [209]. Subsequent work revealed that the responsiveness of the probe can be further improved by the use of a D-ornithine-derived TO-modified PNA monomer, which increased the number of rotatable bonds [210].

**Figure 22:**
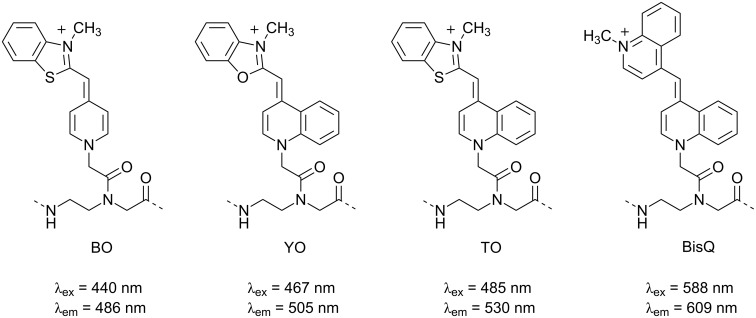
The structures of TO and its analogues that have successfully been used in FIT PNA probes.

Applications of FIT PNA probes for the real-time detection of DNA [210] and RNA [211] by qPCR have been demonstrated. Moreover, FIT PNA probes also recognize dsRNA by triplex formation, yielding an extremely large fluorescence enhancement (>200-fold) that is highly sensitive to base mismatches adjacent to the position of TO, similar to that with ssDNA and ssRNA targets [212–213]. With appropriate cellular delivery techniques, it has been possible to image specific RNA in living cells using FIT PNA probes [214–218]. In addition, FIT PNA probes were shown to offer fast hybridization kinetics, a higher signal-to-background ratio and a better specificity than classical DNA-based molecular beacons. Other related TO derivatives have been explored as alternative fluorophores for FIT PNA probes, but only few close analogs of TO show the selective fluorescence enhancement upon duplex formation that may allow their use in combination with TO-based FIT PNA probes for multiplex detection of DNA or RNA. These include the oxazole yellow (YO) [219], BO [220] and BisQ [221] dyes (Figure 22). Multiplex imaging of two RNAs in living cells was enabled by the combined use of BO- and TO-labeled FIT PNA probes [220]. The wash-free protocol and fast hybridization response of FIT PNA probes allows for both spatial and temporal monitoring of the mRNA expression in the cells [220].

**Figure 23:**
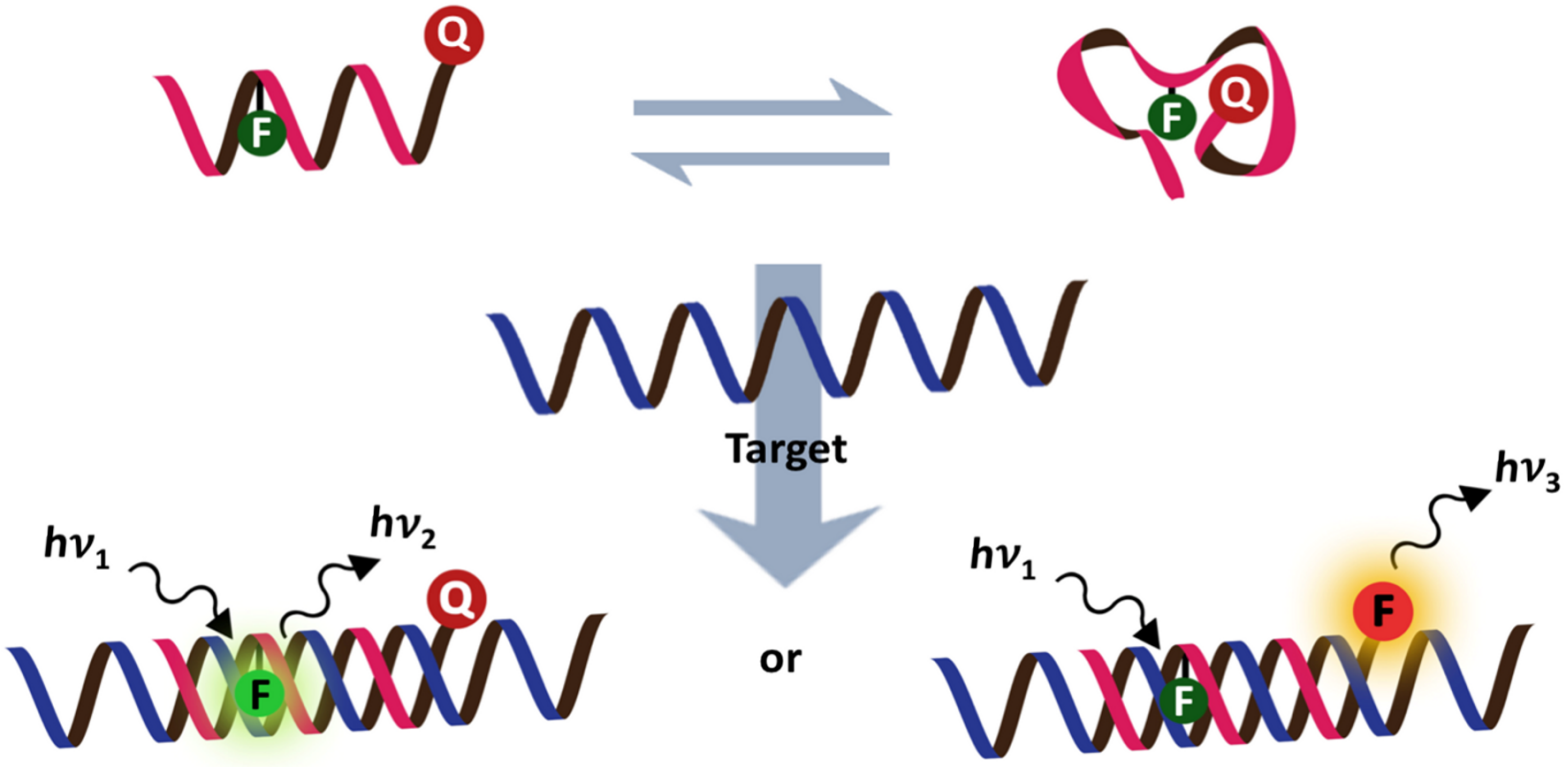
The working principle of dual-labeled FIT PNA probes [222–223].

The performance of FIT PNA probes can be further improved by combination of the TO with an additional dye to form a donor–acceptor pair (Figure 23). This dual-labeled FIT probe design offers advantages over classical stemless PNA beacons with other fluorophore/quencher pairs because the TO dye will also be quenched by the additional dye rather than relying on the rapid internal rotation alone. This results in a much lower background signal of such dual-labeled FIT PNA probes (more than 99.9% lower than single-labeled FIT probes) [222–223]. Upon hybridization, the TO will intercalate and act as a FRET donor provided that the second dye possesses a suitable spectral overlap. Up to a 450-fold enhanced fluorescence emission of the acceptor dyes was observed with TO/indotricarbocyanine (ITCC) FRET pairs (excited at TO and detected at ITCC). On the other hand, if the second dye did not possess a suitable spectral overlap, the dye would simply act as a quencher and the responsiveness of the TO dye was nevertheless still improved as a result of the greatly reduced background signal. Direct excitation of the acceptor dye also showed a markedly improved light-up signal compared to the single-labeled probe due to the mutual quenching of the dye by TO.

In addition to TO, simple polycyclic aromatic hydrocarbons, such as pyrene, have been explored as a non-hydrogen-bonding fluorescence universal base in aegPNA [224–225] and acpcPNA [226]. In the case of aegPNA with triazole-linked pyrene, the duplex stabilities were decreased by 10–12 °C regardless of the nature of the opposite base in the DNA or RNA strand. Hybrid formation resulted in quenching of the pyrene, and stronger quenching was observed with RNA over DNA [225]. The same triazole-linked pyrene in acpcPNA also behaved as a universal base with only slight destabilization of the duplexes. However, the fluorescence change of pyrene-labeled acpcPNA was in the opposite direction to that of aegPNA (light-up instead of quenching) [226]. This was explained by molecular dynamics simulations, which suggested that the triazole formed hydrogen bonds with the opposite nucleobase and pushed the pyrene towards the major groove instead of stacking within the PNA–DNA duplexes [226]. Other chromophores that can potentially behave as non-hydrogen-bonding fluorescent nucleobases in aegPNA include phenylazonaphthalene [227], flavin [228], naphthalimide [229] and psoralen [230]. Fluorescence studies have only been performed with psoralen, and revealed that the fluorescence was only marginally decreased when the PNA probe was hybridized with the complementary DNA target [230].

## Conclusion

This review summarizes various strategies that have been successfully used for the design of fluorogenic PNA probes as well as their performances and applications when applicable. It can be seen that several designs originally developed for oligonucleotide probes can be applied to PNA. The tendency of the uncharged backbone of PNA to fold into a compact structure and the inability to interact with positively charged species that normally bind to nucleic acids offer unique opportunities to design new sensing platforms that have no equivalent in the case of DNA and related analogues. The superior properties of PNA over DNA probes in terms of their improved sensitivity, specificity and/or biological stability have been clearly demonstrated in several cases. Nevertheless, the availability and cost of PNA, together with other unfamiliar characteristics, such as its hydrophobicity, poor water solubility and cellular uptake, make the research community reluctant to adopt PNA as a new tool despite these potential advantages over more conventional oligodeoxynucleotide probes. While it is unlikely that PNA-based fluorogenic probes will generally replace DNA probes for routine applications, there are niche areas that PNA probes can potentially offer real advantages. These include applications that require very high specificity under non-stringent conditions, such as detection of SNPs or closely related nucleic acid targets, especially in multiplex fashion. In addition, PNA probes are especially suitable for targeting nucleic acid targets with secondary structures that may be difficult to access by other probes. The excellent biological stability of PNA-based fluorogenic probes make them particularly attractive as an alternative to oligonucleotide probes for intracellular nucleic acids detection. Although some impressive examples are emerging, most of these are proof-of-principle studies with highly expressed RNA targets. It remains to see if it is possible to increase the sensitivity to detect low abundant targets in situ and in real time. By combination of PNA as a recognition element together with brighter, more stable fluorophores, new signal transduction mechanisms and more sophisticated detection techniques, such as fluorescence lifetime measurement and single molecule fluorescence, there are still a lot of further opportunities for fluorogenic PNA probes to advance the field further.
